# Antioxidant, Anti‐inflammatory, Analgesic, and Antiarthritic Potentialities of Methanolic Extract of Fruit Husk of *Nypa fruticans* Wurmb, Established by In Silico Analysis

**DOI:** 10.1155/bmri/8861594

**Published:** 2026-04-15

**Authors:** Farhana Alam Ripa, Ajoy Paul Adar, Adwiza Chakraborty Bishakha, Nashrah Mustafa, Mehrin Haque Tanisha, Nishat Zareen Khair, Zara Sheikh, Asef Raj, Israt Jahan Bulbul

**Affiliations:** ^1^ School of Pharmacy, BRAC University, Dhaka, Bangladesh, bracu.ac.bd; ^2^ Department of Pharmacy, Southeast University, Dhaka, Bangladesh, seu.edu.bd

**Keywords:** analgesic, anti-inflammatory, antioxidant, in silico analysis, *Nypa fruticans* Wurmb

## Abstract

This study investigates the bioactive components and biological activities of the methanolic husk extract of *Nypa fruticans* Wurmb (MNF) through gas chromatography–mass spectrometry (GC‐MS) analysis. The MNF extract was identified to contain several bioactive components, including alkaloids, glycosides, phenols, tannins, and resins, as established through preliminary phytochemical screening tests. After finding the presence of antioxidant components in the qualitative antioxidative assay, we went to conduct quantitative tests where the total contents of phenolics, flavonoids, and tannins were estimated as 179.43 ± 3.99 mg GAE/g, 179.43 ± 3.99 mg QE/g, and 29.25 ± 0.29 mg TAE/g, respectively. The extract demonstrated notable antioxidant activity in DPPH free radical scavenging (IC_50_ 36.28 *μ*g/mL) and ferric reducing antioxidant power (83.27*%* ± 0.02*%* reduction) assays. The extract demonstrated notable in vitro anti‐inflammatory and antiarthritic properties in a dose‐dependent manner, as indicated by human red blood cell (HRBC) membrane stabilization (74.63*%* ± 0.84*%* at 5000 *μ*g/mL) and protein denaturation (88.77*%* ± 0.31*%* inhibition at 500 *μ*g/mL), relative to standards. The anti‐inflammatory efficacy of MNF extract was confirmed via an in vivo carrageenan‐induced inflammatory study, showing an 85.49% reduction in edema at the maximum dosage of 600 mg/kg. The extract demonstrated significant (*p* < 0.05, *p* < 0.01, and *p* < 0.0001) and dose‐dependent analgesic effects in acetic acid–induced writhing and formalin‐induced pain assays. Molecular docking studies conducted with the selected compounds against potential targets identified 1‐(3,6,6‐trimethyl‐1,6,7,7a‐tetrahydrocyclopenta[c]pyran‐1‐yl) ethanone as the best candidate with promising analgesic and anti‐inflammatory potential, demonstrated by its high binding affinity comparable to or exceeding that of the selected standard drugs. Our findings indicate that *N. fruticans* fruit husk may serve as a viable source of natural chemicals possessing antioxidant, analgesic, anti‐inflammatory, and antiarthritic properties, demonstrating potential therapeutic effects in alleviating diseases associated with inflammatory responses.

## 1. Introduction

Numerous therapeutic plant species have been utilized to safeguard humans against various perilous disorders at certain dosages owing to their bioactive compounds [1]. Therapeutic options are increasingly gaining prominence. The application of medicinal flora for the treatment of several illnesses is captivating [2–4]. Herbal medicine is the main healthcare provider for about four billion developing country residents. Indigenous groups regard herbal therapy as indispensable. Approximately 25% of bioactive compounds that are currently used as prescription drugs have been found to derive from plant sources [5, 6]. Exploring natural pharmaceutical sources may produce new compounds with considerable therapeutic benefits and limited side effects [7].

All organisms inherently generate reactive oxygen species (ROS) as byproducts of aerobic metabolism and several cellular metabolic processes. ROS has the propensity to couple with biological macromolecules, including proteins, lipids, and DNA, to become stable, which can damage proteins and DNA. Sometimes, an excess of ROS can interfere with the body′s defense system against free radical damage, which could lead to oxidative stress (OS) [8]. This scenario leads to a range of illnesses, such as coronary heart disease, neurological disorders, diabetes, arthritis, inflammation, lung damage, and cancer [9–11]. The increasing prevalence of chronic illnesses exacerbated by OS is making phytocompounds with antioxidant properties more important since it is turning into the leading cause of death. Plant‐derived components possess powerful antioxidant properties that can potentially inhibit OS by effectively neutralizing ROS and maintaining redox balance [8–10]. Due to their ability to counteract oxidative damage, natural antioxidants are increasingly being used as a substitute for synthetic antioxidants in food and food supplements. Eating fruits and vegetables high in antioxidants is known to reduce the risk of several diseases brought on by free radicals. Phytochemicals, including polyphenols, carotenoids, and vitamins E and C, are primarily responsible for these health effects [8, 11, 12]. Phenolic compounds are frequently present in edible and inedible herbs, cereals, fruits, vegetables, oils, spices, and other plant materials [12]. However, because endemic plants are only found in specific areas and are only known to the local population, there is a dearth of scientific information regarding the antioxidant capabilities of endemic plants. Consequently, evaluating these attributes continues to be a worthwhile and fascinating undertaking, especially when it comes to identifying potential natural antioxidant sources for functional foods and/or nutraceuticals [11].

Pain, often known as algesia, is defined as an unpleasant sensory and affective experience associated with real or potential tissue damage [13]. The nociceptive system serves a crucial role in our survival by promptly notifying us of potential danger when it detects harmful stimuli. Additionally, the heightened sensitivity associated with inflammatory pain aids in the process of healing and recuperation from injuries [14]. Prostaglandins (PGs), proinflammatory cytokines, and chemokines are substances that promote inflammation and induce pain by directly stimulating nociceptors, which are the main sensory neurons responsible for detecting harmful stimuli [7, 15]. Currently, it is imperative to devise innovative approaches for alleviating pain in order to acquire antinociceptive substances that offer enhanced analgesic effects at reduced doses and minimal side effects [7]. Consequently, it is necessary to seek out plant‐based alternative medicines that might decrease inflammation and pain.

Inflammation typically serves as the body′s natural defense mechanism against many illnesses. However, if the inflammation persists and is untreated, it can have detrimental effects on the body [16]. The pathophysiology of both acute and chronic damage to key organs has been correlated with OS and inflammation [17], and these factors are also important in the molecular pathogenesis of rheumatoid arthritis (RA). RA is a chronic autoimmune ailment characterized by inflammation of the synovial and joint membranes. Additionally, it leads to the deterioration, pain, and distortion of the bone cartilage [18]. Certain autoimmune chronic inflammatory illnesses are commonly treated with immunosuppressors, such as methotrexate, hydroxychloroquine, and sulfasalazine. Chronic inflammatory illnesses are also commonly treated with nonsteroidal anti‐inflammatory drugs (NSAIDs), which block cyclooxygenase‐1 (COX1) and COX2 [19]. Nevertheless, NSAIDs can lead to significant adverse effects or contribute to other medical conditions, including cardiovascular disease, diabetes, and cancer. Several unrefined herbal‐based medications and their components exhibit significant antioxidant properties and effectively eliminate harmful free radicals. These actions contribute to the deterioration of cartilage and the development of inflammatory reactions [18, 20]. Chemical compounds derived from medicinal plants could interact with and regulate the expression of proinflammatory signaling in the inflammation pathway, resulting in a decrease in the arthritic impact [18, 21].

Recently, there has been a notable shift toward researching antioxidants derived from underutilized fruits [22]. These species are characterized by their local availability and global scarcity, yet they remain largely overlooked due to a lack of scientific data and market promotion. Despite possessing vibrant colors and significant health‐promoting properties, many of these nutrient‐dense fruits remain unfamiliar to the general consumer [23, 24].

In recent times, there has been a significant emphasis on conducting research and development efforts focused on antioxidants derived from fruits that are not commonly utilized [22]. They stand out due to their local availability, irregularity on a global scale, and paucity of relevant information. Numerous fruits with varied skin and pulp colors that are beneficial to health can be found. Due to a lack of advertising and marketing, many of these fruits are still unfamiliar.

*Nypa fruticans* Wurmb (Nypa), also referred to as the nipa palm, is a member of the Arecaceae family and is mostly found in tropical regions, which include most of Southeast Asia [25, 26]. In Bangladesh, the natural distribution of nipa palm is restricted to the “Sundarbans,” the largest single continuous tract of mangrove forest in the world. It is a stemless palm with long, upright fronds and a subterranean, rhizomatous stem with a vast root system that is ideal for withstanding quickly flowing water (H_2_O). The leaves are 9–10 m long on average. Plant parts are known to be rich in polyphenols, flavonoids, vitamin E, protocatechuic acid, kaempferol, and calcium [27, 28]. These bioactive compounds are already widely known to have good antioxidative, anti‐inflammatory, and cholesterol‐suppressing effects [26]. Folk remedies have used the roots, leaves, and stems of nipa palm for conditions like pain, diabetes, asthma, tuberculosis, sore throats, and liver disease [22, 28, 29]. A comprehensive investigation of various parts of this plant may be able to provide scientific evidence for its traditional uses. The majority of pharmacological research on the nipa plant was previously done on its leaves, fruits, flowers, and bark; however, very few research has been done on the possible applications of the methanolic extract of the fruit′s husk as an antioxidant, analgesic, anti‐inflammatory, or antiarthritic [30, 31]. In our study, we selected methanol for the extraction of plant constituents due to its superior solubility and efficacy in extracting pharmacologically active compounds, including phenolic compounds, lipids, anthocyanins, and terpenoids [32, 33]. Considering all these facts, we opted to evaluate the aforementioned pharmacological properties of the methanolic extract of nipa fruit husk, acknowledging the plant′s significant concentration of advantageous chemicals and its historical medicinal applications. A computer analysis was conducted to ascertain the precise binding characteristics of the ligands with designated receptors in the human body.

## 2. Materials and Methods

### 2.1. Collection, Identification, and Extraction of Plant Materials

In April 2024, fully mature *N. fruticans* fruits were collected from the Sundarbans mangrove forest located in the Bagerhat Region of Bangladesh. A taxonomist from the Bangladesh National Herbarium in Mirpur, Dhaka, identified the fruits with Accession Number DCAB‐87898. Fruit husks were removed following a comprehensive washing process. The husk was dried for a duration of 1 week under the shade, after which it was pulverized into a fine powder and subsequently preserved in airtight receptacles within a cool, arid, and dimly lit environment till further processing. Subsequently, a quantity of 500 g of the powdered substance was soaked in a glass container containing 2 L of 95% methanol for a duration of 14 days, during which it was intermittently agitated and stirred. After a period of 2 weeks, the entire mixture was condensed with a rotary evaporator (Bibby Sterlin Ltd, United Kingdom) under reduced pressure at 50°C, and then the condensed mixture was passed through a clean cotton bed and Whatman filter paper (No. 1), subjected to freeze‐drying using a benchtop freeze dryer (Virtis, NY, United States), and stored at a temperature range of 4°C to −20°C in a sterile universal container until further analysis [34].

### 2.2. Chemicals and Reagents

The analytical and biological tests were conducted using laboratory‐grade reagents, including methanol, hydrochloric acid, DPPH, gallic acid, ascorbic acid, formalin (Sigma‐Aldrich Ltd, Saint Louis, United States), NaNO_2_, Na_2_CO_3_, Na_2_HPO_4_·2H_2_O, AlCl_3_, dimethyl sulfoxide (DMSO), NaOH, potassium ferricyanide (K_3_Fe(CN)_6_), Folin–Ciocalteu (FC) reagent, quercetin, ferric chloride (FeCl_3_), acetic acid, Alsever′s solution (Merck Group, Darmstadt, Germany), trichloroacetic acid (TCA), and k‐carrageenan (Sigma‐Aldrich Chemicals, India). The procurement of diclofenac sodium and indomethacin was made from Square Pharmaceuticals Ltd., Bangladesh.

### 2.3. Preliminary Phytochemical Screening

The presence of specific bioactive components in the tested extract was verified using standard procedures [35, 36]. Visual examination of color or foaming was used to assess whether a certain phytochemical group was present or absent [36, 37].

### 2.4. Experimental Animals

In vivo experiments were conducted using albino mice (weighing 25–30 g) and Wistar albino rats (weighing 160–250 g). The rodents were acquired from the International Center for Diarrheal Disease Research, Bangladesh (ICDDR,B) Animal Resources Division. The animals were housed in polypropylene cages under controlled laboratory settings (room temperature 25^°^C ± 2^°^C; relative humidity 55%–60%; 12 h light/dark cycle). All rodents were housed in the aforementioned conditions for 10 days in order to allow them to become used to the laboratory setting before beginning any pharmacological studies. This research was conducted in compliance with the International Council for Laboratory Animal Science (ICLAS) and the National Institutes of Health (NIH), which are the internationally recognized authorities on the proper use of laboratory animals. Every protocol for an animal experiment has been approved by Southeast University′s animal ethics committee (SEU/Pharm/CERC/112/2024) in Dhaka, Bangladesh.

### 2.5. In Vitro Antioxidant Tests

#### 2.5.1. Qualitative Antioxidant Test

For this investigation, 1.0 mL of crude extract was combined with 1.0 mL of 0.15 mM DPPH reagent. Antioxidant activity was shown by a color change from purple to yellow. For the subsequent analysis, 1.0 mL of the experimental extract was combined with 1.0 mL of phosphomolybdate reagent. Antioxidant activity was evidenced by the color change from yellow to blue–green [38, 39].

#### 2.5.2. Quantitative Antioxidant Test

a. Measurement of different secondary metabolite content

*Total phenolic content (TPC) measurement*

The TPC of the MNF extract was measured using the FC method, as described by Jahan et al. [40]. Gallic acid was used as a reference for constructing a standard curve. The TPC was quantified as milligrams of gallic acid equivalent (GAE) per gram of dried plant extract.

*Total flavonoid content (TFC) measurement*

The TFC of MNF extract was measured according to the method reported by Jahan et al. [40] using an aluminum chloride colorimetric assay. Quercetin was used as a standard to create a standard curve. The expression of TFC was quantified as milligrams of quercetin equivalent (QE) per gram of dried plant extract.

*Total tannin content (TTC) measurement*

The TTC of MNF extract was measured following the method reported by Debnath et al. [41] using FC reagent and gallic acid as a reference to create a calibration curve. The TTC was quantified in milligrams of tannic acid equivalent (TAE) per gram of dried plant extract.

b. DPPH free radical scavenging assay

The antioxidant potential of the experimental plant extracts was evaluated using the previously described Blois DPPH scavenging assay [41] method, with minor modifications. In short, 3.0 mL of a methanol solution containing DPPH (40 *μ*g/mL) was mixed with different amounts of plant extracts (20–100 *μ*g/mL). Next, using a UV spectrophotometer, the absorbance was determined at 517 nm. The formula used to calculate the free radical scavenging capacity is as follows:

%of inhibition=Ablank−AsampleAblank×100%

The absorbance for each group is denoted by *A*. DPPH radical scavenging activity was quantified as SC_50_ (micrograms per milliliter), which represents the concentration of the sample needed to scavenge 50% of the radical production.

c. Ferric reducing antioxidant power (FRAP) assay

The reduced antioxidant capacity of the tested extract was evaluated using the methodology outlined in Debnath et al. [41], with some modifications. The MNF extract solutions of different concentrations (ranging from 20 to 100 *μ*g/mL) were thoroughly combined with 0.2 M phosphate buffer and 1% K_3_Fe(CN)_6_. The mixture was then incubated at a temperature of 50°C. Subsequently, a mixture of 10% TCA, H_2_O, and 0.1% FeCl_3_ was prepared at each concentration. Next, the absorbance was measured at a wavelength of 700 nm, and the percentage of reduction was determined using the calibration curve. This value was then stated as RC_50_ (micrograms per milliliter), which represents the concentration of the sample necessary to decrease 50% of Fe^3+^.

### 2.6. Acute Toxicity Study

According to OECD (2008), 425 normal, mature, and nonpregnant female Swiss Albino mice were tested for acute oral toxicity [38]. Hence, five 25–30 g female mice were utilized. All animals were fasted for 3 h before receiving the plant extract. After fasting, one female mouse received a single oral limit test dosage of 2 g/kg of the test extract. For 4 h, every 30 min, the mouse was monitored for toxicity and mortality within 24 h. The first mouse′s results were used to give the next four mice an equivalent dose of the extract in succession. The mice were then housed individually and examined for 4 h with a 30‐min break and daily for 2 weeks for toxicity‐related behavioral, autonomic, neurologic, and physical abnormalities [42].

### 2.7. In Vitro Anti‐Inflammatory Assay

The human red blood cell (HRBC) membrane stabilization method plays a major role in determining the anti‐inflammatory activity of plant extracts [43]. A freshly taken human blood sample was mixed with an equivalent volume of sterilized Alsever′s solution (2% dextrose, 0.8% sodium citrate, 0.5% citric acid, and 0.42% sodium chloride in H_2_O). The participant in the sample abstained from taking NSAIDs for a duration of 2 weeks before the collection of blood samples. The collected blood was centrifuged for 10 min at 3000 rpm, after which the pellet, or packed cells, was rinsed three times with isosaline (0.85%, pH 7.2), and finally, a 10% (*v*/*v*) solution was made using isosaline. The experimental setup involved adding 1 mL of a phosphate buffer solution with a concentration of 0.15 M and a pH of 7.4, along with 2 mL of a hyposaline solution with a concentration of 0.36%. Additionally, 0.5 mL of an HRBC suspension was added to the different doses of plant extract. Extracts were omitted from the standard or control preparation. The standard drug consisted of different concentrations of indomethacin (1000, 2000, 3000, 4000, and 5000 *μ*g/mL), which were compared to corresponding quantities of plant extract. Following a 30‐min incubation at a temperature of 37°C, the reaction mixtures were subjected to centrifugation for 10 min at a speed of 3000 rpm. The optical density (OD) of the supernatant was measured at 560 nm.

The percentage hemolysis was calculated using the following equation:

%of Hemolysis=ODtest−ODcontrolODcontrol×100%

The HRBC membrane stabilization percentage was determined using the following equation:

%of Protection=100−ODtest−ODcontrolODcontrol×100%

### 2.8. In Vivo Anti‐Inflammatory Assay

Carrageenan‐induced paw edema in Wister albino rats was used to assess the MNF extract′s in vivo anti‐inflammatory activity [44, 45]. A plethysmometer was employed to quantify the decrease in inflammation. To induce inflammation, a 1% solution of carrageenan (100 *μ*L in physiological saline) was administered subcutaneously to the right hind paw 1 h after treatment administration (normal saline 5 ml/kg, PO), indomethacin (15 mg/kg, ip), and varying doses of the plant extract (−200, 400, and 600 mg/kg, orally). The alteration in paw dimensions (measured in millimeters) was once again assessed following the induction of inflammation, starting at 0 h and continuing for a duration of 5 h. The percentage inhibition of paw edema was calculated using the following formula:

%Inhibition of paw edema=Vc−VtVc×100

where *V*_*c*_ and *V*_*t*_ represent the average paw volume of the control and treated animals, respectively.

### 2.9. In Vivo Analgesic Activity

The extract was tested for analgesia using acetic acid–induced peripheral writhing and central formalin‐induced nociception (pain) models.

#### 2.9.1. Peripheral Analgesic Test (Acetic Acid–Induced Writhing Method)

The acetic acid writhing test was employed to evaluate the peripheral analgesic efficacy, following previous studies with minor amendments [46, 47]. Thirty mice weighing between 20 and 30 g were split into five groups (Groups I–V) at random (*n* = 6). The control group (Group I) received normal saline (5 mL/kg, ip), the standard group (Group II) received indomethacin (15 mg/kg, oral), and the treated groups (Groups III–V) received MNF extract orally at varied doses (200, 400, and 600 mg/kg). An injection of 0.1 mL of acetic acid (0.6% *v*/*v*) was administered intraperitoneally 60 min after the treatments were given. The cumulative count of abdominal muscle contractions, known as writhes, was recorded for a duration of 5–30 min following the administration of acetic acid. To assess the analgesic activity, we calculated the average number of writhes and the percentage of writhing inhibition [46, 47]:

%of writhing inhibition=Ncontrol−NtestNcontrol×100%

Here, *N* represents the mean number of writhing for each group.

#### 2.9.2. Formalin Nociceptive Assay

The antinociceptive property was evaluated using the formalin‐induced pain technique in the Swiss albino mouse model, following the protocol described by Islam et al. [48] with minor adjustments. Plant extract (various doses) and standard are typically given 60 min prior to the subcutaneous formalin injection. Following this, 5 *μ*L of formalin (2.5% formaldehyde) was injected into the plantar surface of the mouse hind paw. After the formalin injection, the mice were observed for a period of 30 min, during which the amount of time they spent gripping the injected hind paw was recorded. “Early phase” refers to the first 5 min after the injection of formalin, and “late phase” refers to the 15–30‐min period that follows. Timed intervals of swishing or nibbling the wounded paw (pain behavior) were recorded for every animal using a stopwatch.

### 2.10. In Vitro Antiarthritic Assay by Protein Denaturation Method

The antiarthritic efficacy of MNF extract was studied using the protein denaturation methodology, following the method described by Ripa et al. and Chikowe et al. with little amendment [49, 50]. The reaction combination for the test solution (0.5 mL) included 5% *w*/*v* bovine albumin (0.45 mL) and test sample (0.05 mL), while the control solution (0.5 mL) included 0.45 mL bovine serum albumin (BSA) (5% *w*/*v*) and 0.05 mL distilled H_2_O. MNF extract and diclofenac sodium were tested at various concentrations (100, 200, 300, 400, and 500 *μ*g/mL). After adjusting the pH to 6.3 with 1N HCl, the solutions were incubated at 37°C for 20 min. All solutions were then kept in the incubator at 57°C for an additional 30 min. Following the incubation period, all solutions were allowed to cool before being mixed with 2.5 mL of phosphate buffer. The UV‐visible spectrophotometer was used to measure the absorbance of the solutions at a wavelength of 660 nm. The control exhibits complete protein denaturation. The equation employed to quantify the degree of protein denaturation inhibition was as follows:

%of inhibition=Acontrol−AtestAcontrol×100%

where *A* represents the absorbance at 660 nm for each group.

### 2.11. GC‐MS Analysis

GC‐MS is considered a credible biophysical method for the identification of phytochemical screening of plant extracts. GCMS‐TQ8040 was employed for identifying the compounds in the fraction we analyzed. The column was DB‐5ms (30 m, 0.25 mm ID, 0.25 *μ*m df), and helium was used as the carrier gas. The temperature for the injection was chosen at 250°C. The initial temperature of the oven was 40°C, which was maintained for 2 min, and after that, the temperature reached 200°C at the rate of 15°C/min, which was again maintained for 2 min. This temperature finally reached 300°C with a hold time of 7 min and a rate of 5°C/min. The ion source temperature was 230°C. The injection mode was set in the splitless mode with a split ratio of 1:50. The detector gained the ionizing energy at 0.50 kV for the detection of GC‐MS. With a scan interval of 0.300 s, the gas chromatography continued for 36.5 min. We interpreted the mass spectrum of prepared unknown compounds by comparing them to the known compounds restored in the library of the National Institute of Standards and Technology (NIST), 2008 and 2014 editions [51].

### 2.12. In Silico Study

#### 2.12.1. Ligand Preparation

The most abundant compounds identified from the extract were selected for further molecular docking analysis. Three‐dimensional structures of these compounds were retrieved in SDF format from the PubChem database. In addition to these compounds, several standard drugs—including indomethacin‐(R)‐alpha‐ethyl‐ethanolamide (IM8), SC‐558, BU72, NADPH, xanthene, SN37333, BPTES, and glutamate—were also downloaded from PubChem and included as reference ligands.

Conformational analysis of all ligands was performed manually by rotating freely rotatable bonds to generate possible conformers. These conformers were then optimized using the general AMBER force field (GAFF) via the “optimize geometry” function in Avogadro 1.2. Ligand structures were further prepared for docking using AutoDock Tools (Version 1.5.6), where Gasteiger charges were assigned, and the structures were kept flexible to allow for multiple binding poses.

#### 2.12.2. Protein Preparation

Protein structures were retrieved from the RCSB PDB database (https://www.rcsb.org/) and selected based on their x‐ray diffraction method and high‐resolution data. All selected protein structures were subsequently evaluated against 23 compounds identified through GC‐MS analysis.

For the assessment of analgesic activity, protein molecules including COX1 (PDB ID: 2OYE), COX2 (PDB ID: 6COX), and the Mu opioid receptor (PDB ID: 5C1M) [52] were sourced from the Protein Data Bank. Human erythrocyte catalase (PDB ID: 1DGH) and human glutathione reductase (GR) (PDB ID: 1XAN) were selected to evaluate antioxidant potential. To investigate anti‐inflammatory activity, human MR1 (PDB ID: 5UR1) and aldo‐ketoreductase family 1‐member C3 (AKR1C3) (PDB ID: 1S1P) [53] were included, while glutaminase 1 (GLS1) (PDB ID: 3VP1) was chosen for assessing antiarthritic activity [54]. All H_2_O molecules, cofactor (FAD), and ligand (harmine) were removed from the receptor protein by using PyMOL software.

#### 2.12.3. Absorption, Distribution, Metabolism, and Excretion (ADME) Prediction

The physicochemical properties, pharmacokinetic behavior, drug‐likeness, and medicinal chemistry profiles of the phytocompounds were evaluated using the online tool QikProp (Schrödinger) (https://www.schrodinger.com/platform/products/qikprop/) [55]. Compounds were screened for their potential as drug candidates based on Lipinski′s rule of five, which assesses oral bioavailability by evaluating key molecular parameters. According to Lipinski, a compound is likely to exhibit drug‐like behavior if it meets at least four out of five criteria: molecular weight (MW) ≤ 500, no more than 10 hydrogen bond acceptors, no more than five hydrogen bond donors, a lipophilicity (log Po/w) less than 5, and a molar refractivity in the range of 40–130. Selected compounds that satisfied these criteria were considered promising drug candidates and were further evaluated for their ADME properties.

#### 2.12.4. Molecular Docking

Out of 82 phytocompounds identified through GC‐MS analysis, 23 compounds were selected for molecular docking based on their percentage abundance greater than 1%. The selected compounds were then docked against various protein targets: COX1 (PDB ID: 2OYE), COX2 (PDB ID: 6COX), the Mu opioid receptor (PDB ID: 5C1M), human erythrocyte catalase (PDB ID: 1DGH), human GR (PDB ID: 1XAN), human MR1 (PDB ID: 5UR1), AKR1C3 (PDB ID: 1S1P), and GLS1 (PDB ID: 3VP1).

Molecular docking was performed using PyRx (Version 0.8) with AutoDock Vina [56]. Protein structures were treated as macromolecules, and the ligands were converted into PDBQT format. Grid boxes were defined to encompass the active site residues of respective proteins, ensuring optimal ligand binding poses. Binding affinities were calculated and reported in kilocalories per mole. For each ligand, PyRx generated up to five different docking poses and calculated the corresponding binding affinity values. The program evaluated the protein–ligand complex affinity energy based on the ligand conformations at the active site, also considering the root mean square deviation (RMSD) between the original and docked structures. The complex with the lowest binding energy (i.e., the highest binding affinity) was selected for further analysis.

Amino acid residues involved in ligand binding, bond types, and bond lengths were identified for each protein–ligand complex. These interactions were further visualized and analyzed using Discovery Studio, which provided detailed representations of both covalent and noncovalent interactions. A comparative analysis was performed to identify common amino acid residues consistently involved in ligand binding across all selected compounds.

### 2.13. Molecular Dynamics Simulation

MD simulation was conducted using the three most potential molecules obtained from molecular docking for a duration of 100 ns. The compound–protein complexes, 1‐(3,6,6‐trimethyl‐1,6,7,7a‐tetrahydrocyclopenta[c]pyran‐1‐yl) ethanone with 1S1P, 4‐hydroxy‐1H‐pyrazolo [3,4‐D] pyrimidine riboside with 1XAN, and 1‐(3,6,6‐trimethyl‐1,6,7,7a‐tetrahydrocyclopenta[c]pyran‐1‐yl) ethanone with COX2 complexes, were used. The AMBER14 force field [57] was utilized to perform simulation using the YASARA Dynamics product (http://www.yasara.org/products.htm) in the YASARA program (http://www.yasara.org/index.html) Version 23.9.29.W.64 for the Windows environment software with a timestep of 2.50 fs [58]. A cubic simulation cell was constructed with periodic boundary conditions, ensuring dimensions at least 20 Å larger than the complex. The system was kept at a physiological pH of 7.4 and a temperature of 298 K, with a pressure of 1 atm, controlled through the Berendsen thermostat [59]. The system was neutralized through the introduction of H_2_O molecules at a density of 0.997 g/cm^3^, along with NaCl at a concentration of 0.9%, through the TIP3P (transferable intermolecular interaction potential 3 points) method [57, 60]. MD trajectories were collected every 250 ps for postsimulation analysis. The trajectory files were examined following the 100 ns simulation in order to ascertain the RMSD, root mean square fluctuation (RMSF), solvent‐accessible surface area (SASA), radius of gyration (Rg), and H‐bond analysis scores. Therefore, the MM‐PBSA (molecular mechanics Poisson–Boltzmann surface area) method was employed to determine the binding free energy based on the trajectory files, with a higher positive energy value signifying a more advantageous binding interaction. The formula that was used to determine the binding free energy is as follows [61]:

Binding Free Energy=Epot Recept+Esolv Recept+Epot Ligand+Esolv Ligand−Epot Complex−Esolv Complex

### 2.14. Statistical Analyses

The results are expressed as mean ± SEM. Statistical analysis utilized one‐way and two‐way analysis of variance (ANOVA), accompanied by Dunnett′s post hoc test, except for the in vitro anti‐inflammatory and antiarthritic tests with SPSS 26 software. All cases were deemed statistically significant at *p* < 0.05. The graphs presented in this manuscript were generated using GraphPad Prism 11 software (GraphPad Software Inc., San Diego, CA).

## 3. Result

### 3.1. Preliminary Phytochemical Screening

The results of the phytochemical screening of *N. fruticans* husk extract are compiled in Table 1. The crude methanolic extract exhibited a variety of advantageous secondary metabolites. All examined phytochemicals were identified in the MNF extract. Fixed oil was the least prevalent secondary metabolite among all the bioconstituents.

**Table 1:** Phytochemical screening test of *N. fruticans* husk extract.

Phytocompounds	Test name	Observation	MNF
Glycoside	Borntrager′s test	The ammoniacal layer turned pink.	++
Carbohydrate	Molisch′s test	A reddish‐violet ring was observed at the interface between the acid and the sample in Molisch′s test.	+++
Resin	Ferric chloride test	The solution color was changed to greenish blue.	++
Alkaloid	Mayer′s test and Dragendorff′s test	The creamy white‐colored precipitate was noticed in Mayer′s test, while the orange–red precipitate was observed in Dragendorff′s test.	++
Saponin	Frothing test	Formation of foam on the top of the test tubes.	+++
Tannin	Gelatin test	Precipitate formed due to the addition of gelatin.	++
Steroid	Liebermann–Burchard′s test	The upper layer turned red, and the sulfuric acid layer turned yellow–green in the test tube.	++
Flavonoid	Shinoda′s test	The solution changed to a pink color.	+++
Phenol	Ferric chloride test	The color of the extract solution changed to violet.	+++
Fixed oil	Stain test	A translucent spot or stain was observed on the paper.	+
Terpenoids	Salkowski′s test	A reddish‐brown color at the interface between the two liquids was observed.	++

*Note:* Here, “+” states the presence shows the absence of any phytochemical group. Bioavailability key: (+++) ve = robust intensity, (++) ve = medium intensity, (+) ve = poor intensity.

### 3.2. In Vitro Antioxidant Tests

#### 3.2.1. Qualitative Antioxidant Test

The qualitative DPPH antioxidant assay indicated a color change of the extract from purple to yellow–brown, confirming the antioxidant properties of the tested material. In an additional antioxidant assay (phosphomolybdate reduction test), the crude extract demonstrated a positive outcome by altering its color from yellow to greenish blue (Table 2).

**Table 2:** The qualitative tests for antioxidant activity with DPPH and phosphomolybdate.

Tests	Extract	Result
Antioxidant with DPPH	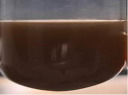	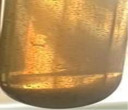
Antioxidant with phosphomolybdate	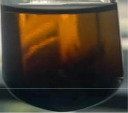	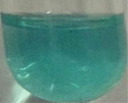

#### 3.2.2. Quantitative Antioxidant Test

a. Measurement of different secondary metabolite content

All data are given in Supporting Information 2: File S2. Following favorable qualitative antioxidant assessments of the MNF extract using DPPH and phosphomolybdate (Table 2), we conducted quantitative analyses of several antioxidant constituents. The total amounts of phenolics, flavonoids, and tannins were evaluated against gallic acid or quercetin using standard techniques. All data are given in Supporting Information 2: File S2. The MNF extract demonstrated total concentrations of phenolics, flavonoids, and tannins at 179.43 ± 3.99 mg GAE/g, 181.69 ± 0.92 mg QE/g, and 29.25 ± 0.29 mg TAE/g, respectively (Table 3).

b. DPPH free radical scavenging assay

**Table 3:** Total phenolic contents (TPCs), total flavonoid contents (TFCs), and total tannin contents (TTCs) of the MNF extract.

Plant extract	Total phenolic content (mg GAE/g)	Total flavonoid content (mg QE/g)	Total tannin content (mg TAE/g)
MNF	179.43 ± 3.99	181.69 ± 0.92	29.25 ± 0.29

*Note:* Data are expressed as means ± standard deviation (*n* = 3).

The antioxidant efficacy of husk extract has been examined through its capacity to diminish DPPH levels. The interaction of antioxidant chemicals with DPPH involves the transfer of a hydrogen atom or electron to the DPPH radical, resulting in its conversion to 1,1‐diphenyl‐2‐picrylhydrazine. The decrease of DPPH radicals results in a color change from purple to pale yellow, indicating scavenging activity [49]. Data are given in Supporting Information 2: File S2. The antioxidant efficacy of the MNF extract and ascorbic acid against the DPPH assay was evaluated at doses between 20 and 100 *μ*g/mL, as presented in Table 4. Based on the calculated IC_50_ value, the extract demonstrated significant DPPH scavenging activity (IC_50_ 39.45 *μ*g/mL) when compared to the standard ascorbic acid (IC_50_ 36.28 *μ*g/mL).

c. FRAP assay

**Table 4:** Antioxidant activity of MNF extract with DPPH.

Conc. (*μ*g/mL)	MNF extract		Ascorbic acid	
	% of inhibition	IC_50_ (*μ*g/mL)	% of inhibition	IC_50_ (*μ*g/mL)
20	51.19 ± 0.01	39.45	54.52 ± 0.00	36.28
40	63.09 ± 0.02		66.90 ± 0.01	
60	70.24 ± 0.02		72.62 ± 0.00	
80	75.48 ± 0.03		77.00 ± 0.01	
100	86.19 ± 0.03		91.19 ± 0.01	

*Note:* Data are expressed as means ± SD (*n* = 3).

**Figure 1:**
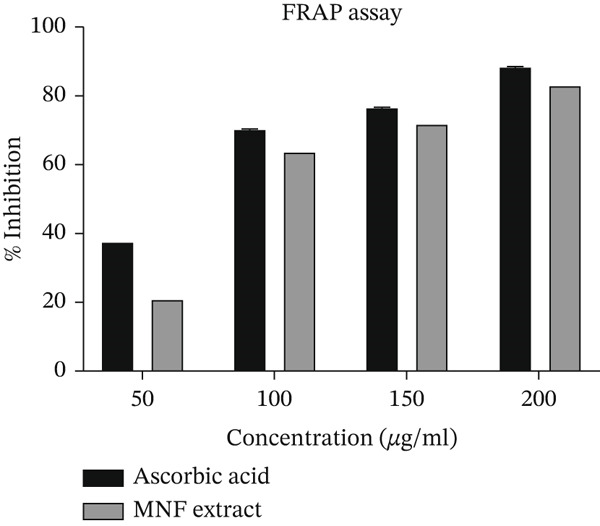
Ferric reducing antioxidant power (FRAP) assay. Percentage of inhibition results of MNF extract compared to standard ascorbic acid. Values are expressed as mean ± SD (*n* = 3).

This study indicates that antioxidants in the sample facilitate the reduction of ferricyanide Fe^3+^ to ferrocyanide Fe^2+^ through electron donation. The antioxidant activity of the sample induced a color shift in the solution from yellow to pale. The methanolic extract of the husk of *N. fruticans* was analyzed using the FRAP assay in conjunction with standard ascorbic acid. Data are given in Supporting Information 2: File S2. The ferric reduction capacity of the examined methanolic plant sample is illustrated in Figure 1. The normal L‐ascorbic acid demonstrated 88.95*%* ± 0.04*%* activity at a concentration of 200 *μ*g/mL, while the MNF extract displayed 83.27*%* ± 0.02*%* at the identical concentration. This indicates that the reducing capacity of MNF extract is comparable to that of the standard ascorbic acid.

### 3.3. Acute Toxicity Study

According to the study, throughout the first 24 h and the next 14 days, the MNF extract did not result in any mortality at a single limit test dose of 2000 mg/kg. Furthermore, the toxicity analysis revealed no significant indicators of overt toxicity, including behavioral, psychiatric, somatic, or physical abnormalities (the experimental mice exhibited no changes in their eyes, skin color, fur, or mucous membranes during the examination).

### 3.4. In Vitro Anti‐Inflammatory Assay

Figure 2 shows the membrane stabilization experiment results. MNF extract showed concentration‐dependent anti‐inflammatory activity compared to indomethacin. Data are given in Supporting Information 2: File S2. The maximum concentration tested was 5000 *μ*g/mL. At the highest dose, indomethacin showed 80.88*%* ± 1.33*%* protection, whereas the MNF extract showed 74.63*%* ± 0.84*%* protection (Figure 2b).

Figure 2(a) Percentage of hemolysis and (b) percentage of protection for indomethacin and MNF extract. Here, data are presented as mean ± SD (*n* = 3), and two‐way ANOVA tests followed by post hoc comparisons were done for the statistical analysis, where the significance levels are as follows: ns = not significant;  ^∗^ *p* < 0.05 and  ^∗∗^ *p* < 0.01.

**Figure:**
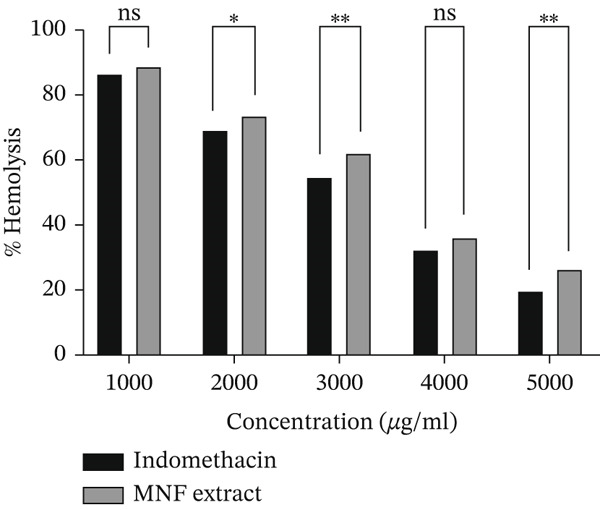
(a)

**Figure:**
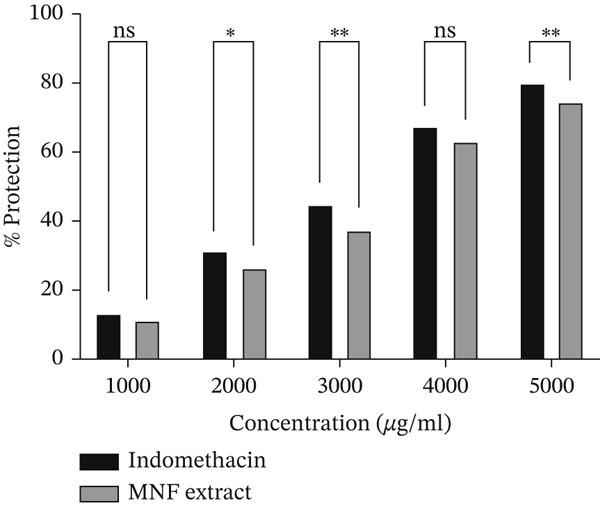
(b)

### 3.5. In Vivo Anti‐Inflammatory Assay

The administration of MNF extract markedly decreased the average paw volume in rodents. All data are given in Supporting Information 2: File S2. The anti‐inflammatory efficacy of the tested extract was assessed using carrageenan‐induced inflammation in rats. The intraplantar administration of carrageenan significantly augmented the thickness of the rat paw. MNF extract significantly (*p* < 0.05 and *p* < 0.01) decreased paw inflammation in a dose‐dependent fashion. MNF at 600 mg/kg exhibited a significant inhibition percentage of 85.49% at the fifth hour of assessment, in contrast to the 93.73% achieved by the standard medication (Table 5). The degree of significance was determined by using a Dunnett test following a one‐way ANOVA for the multiple comparison test.

**Table 5:** Carrageenan‐induced anti‐inflammatory activity of MNF extract.

Treatment	Mean edema volume (mL) and inhibition (%)				
	1 h	2 h	3 h	4 h	5 h
Control	0.178 ± 0.007	0.203 ± 0.010	0.465 ± 0.015	0.495 ± 0.005	0.193 ± 0.005
Diclofenac Na	0.101 ± 0.009 (43.82%) ^∗∗^	0.075 ± 0.005 (63.05%) ^∗∗^	0.071 ± 0.004 (84.30%) ^∗^	0.036 ± 0.005 (92.73%) ^∗^	0.013 ± 0.005 (93.73%) ^∗^
MNF 200	0.156 ± 0.008 (12.36%) ^∗^	0.140 ± 0.008 (31.03%) ^∗^	0.135 ± 0.005 (70.97%)	0.101 ± 0.007 (79.59%) ^∗^	0.068 ± 0.007 (64.76%) ^∗^
MNF 400	0.135 ± 0.005 (24.16%) ^∗^	0.111 ± 0.004 (45.32%)	0.106 ± 0.005 (77.20%) ^∗^	0.088 ± 0.009 (82.22%) ^∗∗^	0.048 ± 0.005 (79.27%)
MNF 600	0.118 ± 0.004 (33.71%) ^∗∗^	0.095 ± 0.008 (52.20%) ^∗∗^	0.09 ± 0.006 (80.64%) ^∗∗^	0.053 ± 0.005 (89.29%) ^∗∗^	0.031 ± 0.004 (85.49%) ^∗∗^

^∗^ (*p* < 0.05) and  ^∗∗^ (*p* < 0.01) denote a significant reduction in paw edema volume relative to the control value for the corresponding time interval (one‐way ANOVA for multiple comparisons followed by Dunnett′s test).

### 3.6. Analgesic Activity

#### 3.6.1. Acetic Acid–Induced Writhing Method

**Figure 3:**
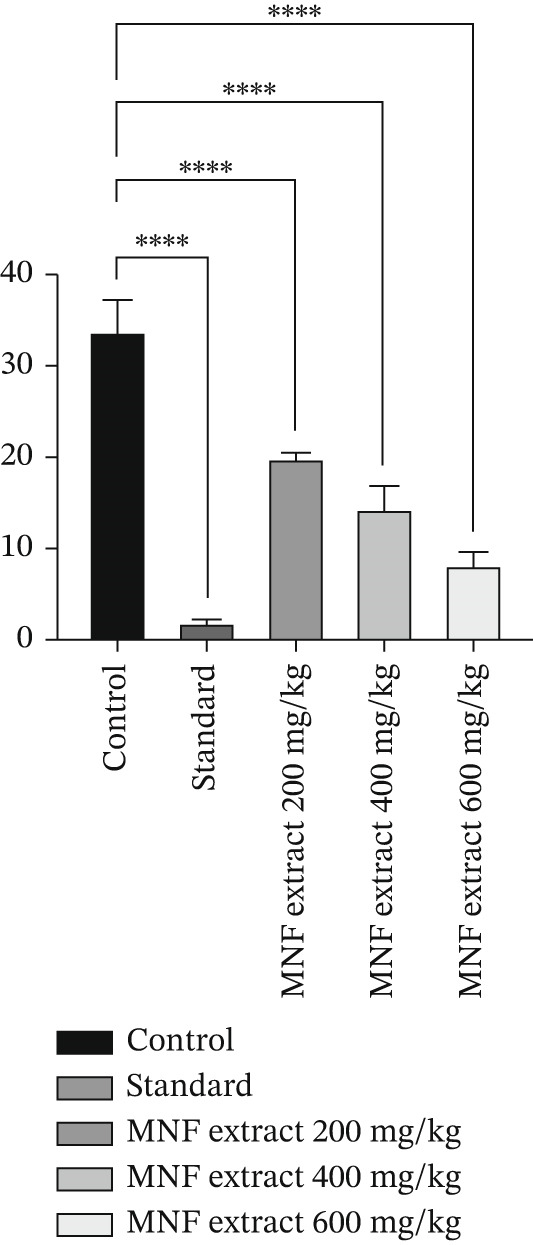
Effect of MNF extract on acetic acid–induced writhing in mice. Here, data are presented as mean ± SEM (*n* = 6), and  ^∗∗∗∗^ means a significant difference with the control group: *p* < 0.0001.

Indomethacin reduced writhing by 94.54%, a statistically significant result (*p* < 0.0001) compared to the control group. All data are given in Supporting Information 2: File S2. Conversely, MNF extract (200, 400, and 600 mg/kg) dramatically reduces writhing in mice (*p* < 0.0001) in a dose‐dependent manner compared to the control (Figure 3). The extract administered at dosages of 200, 400, and 600 mg/kg resulted in reductions of writhing by 41.28%, 57.21%, and 76.12%, respectively. Compared to the reference drug, our results show that the extract has a discernible ability to prevent the synthesis of pain mediators.

#### 3.6.2. Effect of MNF Extract on Formalin‐Induced Pain

The duration of licking in seconds was utilized to assess analgesic efficacy (formalin technique), both shortly after and later following the administration of conventional indomethacin and MNF extract. In the inflammatory phase, paw licking duration was significantly reduced compared to the neurogenic phase, indicating that the plants may mitigate inflammatory pain. All data are given in Supporting Information 2: File S2. Reaction times for licking and biting the rear paws diminished with escalating doses (from 200 to 600 mg/kg), exhibiting a significant reduction (*p* < 0.05 and *p* < 0.01) in the late phase for both the plant extract and conventional indomethacin. In the initial phase, doses of 200, 400, and 600 mg/kg decreased licking time by 37.65%, 50.61%, and 63.56%, respectively. During the final phase, percentage protection values ranged from 37.75% to 62.91% (Table 6). Indomethacin and MNF extract at a 600 mg/kg dose showed a greater analgesic effect, particularly in the early stages.

**Table 6:** Effect of MNF extract on the formalin‐induced paw licking test.

Treatment	Early phase (sec)	Late phase (sec)	% protection of the early phase	% protection of the late phase
Control	41.17 ± 0.75	50.33 ± 1.21	—	—
Indomethacin 10 mg/kg	11.67 ± 0.82^∗∗^	16.17 ± 1.94^∗∗^	71.66	67.88
MNF 200 mg/kg	25.67 ± 2.16^∗∗^	31.33 ± 1.63^∗^	37.65	37.75
MNF 400 mg/kg	20.33 ± 1.21^∗∗^	26.67 ± 0.81^∗^	50.61	47.02
MNF 600 mg/kg	15.00 ± 0.89^∗∗^	18.67 ± 1.03^∗∗^	63.56	62.91

*Note:* The values are revealed as mean ± SD (*n* = 6) and one‐way analysis of variance (ANOVA) followed by Dunnett′s test.

^∗^ *p* < 0.05 and  ^∗∗^ *p* < 0.01 are significant compared to the control.

### 3.7. In Vitro Antiarthritic Assay

**Figure 4:**
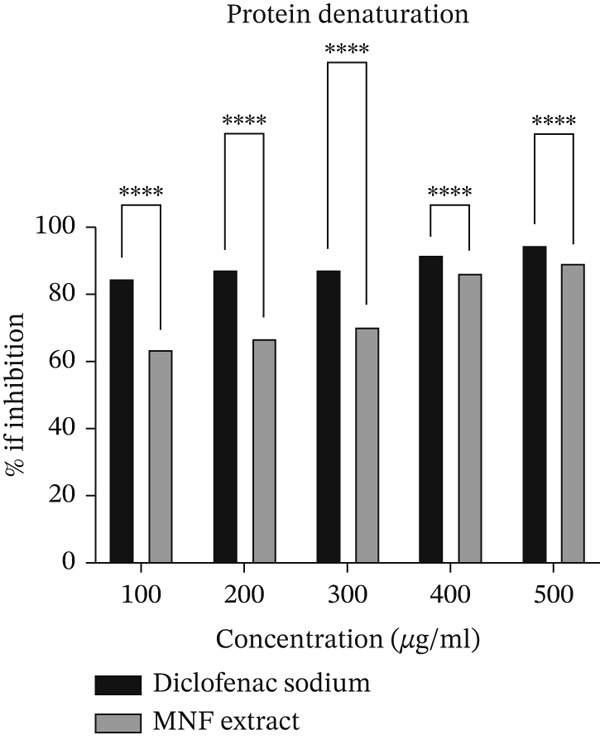
Percentage of inhibition of diclofenac sodium and MNF extract (two‐way ANOVA). Here, data are presented as mean ± SD (*n* = 3), and two‐way ANOVA tests followed by post hoc comparisons were done for the statistical analysis, where the significance levels are as follows:  ^∗∗∗∗^ *p* < 0.0001, which means results are extremely significant when the standard and MNF extracts were compared.

At five different concentrations (100, 200, 300, 400, and 500 *μ*g/mL), the analyzed extract′s antiarthritic efficacy was assessed using an antidenaturation assay based on BSA. Data are given in Supporting Information 2: File S2. Figure 4 displays all of the findings. The plant extract was observed to inhibit BSA denaturation in a concentration‐dependent manner. Compared to the standard medication (94.77*%* ± 0.09*%*), MNF exhibited a significantly higher maximum percentage inhibition (88.77*%* ± 0.31*%*) at a concentration of 500 *μ*g/mL. In all doses, the experimented extract was found to be extremely significant (*p* < 0.0001) when comparing the inhibition percentage to the standard drug diclofenac sodium. This extremely significant (*p* < 0.0001) result indicates a high confidence that the plant extract has a real, reproducible, and effective antiarthritic mechanism.

### 3.8. GC‐MS Analysis

**Figure 5:**
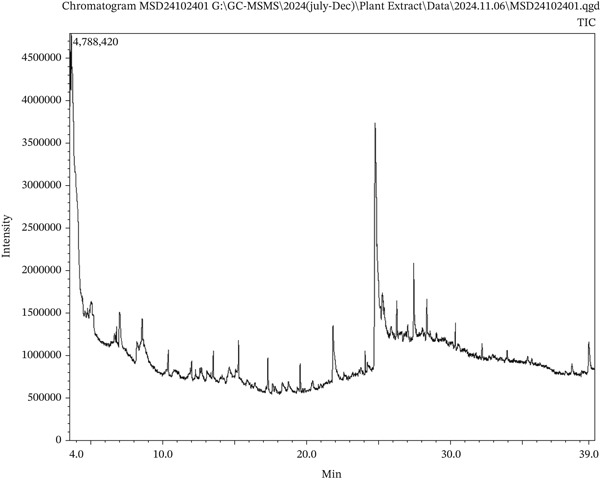
GC‐MS chromatogram of MNF extract.

The GC‐MS analysis detected a total of 82 substances (Supporting Information 1: File S1, Table 1), which are categorized into numerous classes, including fatty acids, esters, phenolic compounds, and terpenoids. These phytochemical compounds from MNF extract were found by employing mass spectrum analysis and retention time (Figure 5).

### 3.9. Molecular Docking Results

To assess the interaction and molecular contacts between target proteins and selected ligands, molecular docking studies were performed using various protein–ligand systems corresponding to analgesic, antioxidant, anti‐inflammatory, and antiarthritic activities. Out of the 82 compounds obtained from GC‐MS, 23 compounds were selected for docking based on their percent abundance greater than 1%, as shown in Supporting Information 1: File S1 (Table 1). Based on the binding affinity with the respective target proteins, three compounds, 1‐(3,6,6‐trimethyl‐1,6,7,7a‐tetrahydrocyclopenta[c]pyran‐1‐yl) ethanone, 4‐hydroxy‐1H‐pyrazolo [3,4‐D] pyrimidine riboside, and maltose, were found to possess promising anti‐inflammatory, analgesic, and antioxidant activity. These three compounds showed binding affinities comparable to or better than their respective standard drugs. Their binding affinity, along with the key amino acid residues they interact with, is shown in Table 7.

**Table 7:** Binding affinity and amino acid residue contacts of ligand–protein complexes.

Activity	Protein PDB ID	Ligand	Binding affinity (−kcal/mol)	Amino acid residue contacts
Analgesic	2OYE	Indomethacin (standard drug)	−9.1	MET522, ILE523, SER353, TYR355, LEU93, VAL116, LEU357, HIS90, VAL349, LEU352, ALA527, and LEU359
		1‐(3,6,6‐Trimethyl‐1,6,7,7a‐tetrahydrocyclopenta[c]pyran‐1‐yl) ethanone (PubChem ID: 605654)	−7.4	ILE523, VAL349, VAL116, ARG120, ALA527, LEU531, VAL116, VAL349, LEU359, LEU531, LEU352, TYR355, and PHE518
		4‐Hydroxy‐1H‐pyrazolo[3,4‐D] pyrimidine riboside (PubChem ID: 135407110)	−7.4	HIS388, THR206, ASN382, TYR385, and GLN203
	6COX	Indomethacin (standard drug)	−6.3	THR94, HIS95, ASP515, and ALA516
		1‐(3,6,6‐Trimethyl‐1,6,7,7a‐tetrahydrocyclopenta[c]pyran‐1‐yl) ethanone (PubChem ID: 605654)	−8	ARG120, TYR355, VAL349, LEU352, ALA527, and VAL523
	5C1M	Indomethacin (standard drug)	−8.5	TYR148, ILE322, ASP147, SER55, HIS54, ILE296, TRP293, TYR326, and VAL300
		4‐Hydroxy‐1H‐pyrazolo[3,4‐D] pyrimidine riboside (PubChem ID: 135407110)	−7.3	TRP318
Antioxidant	1DGH	Ascorbic acid (standard drug)	−6.5	ARG72 and SER114
		Maltose (PubChem ID: 439186)	−8.6	VAL74, ILE332, GLY147, ARG72, TYR358, ARG365, and HIS362
	1XAN	Ascorbic acid (standard drug)	−6.3	VAL329, SER30, GLY157, and ASP331
		4‐Hydroxy‐1H‐pyrazolo[3,4‐D] pyrimidine riboside (PubChem ID: 135407110)	−8.6	ALA155, GLU50, THR57, ASP331, THR156, and GLY56
Anti‐inflammatory	5UR1	Diclofenac sodium (standard drug)	−7.6	GLU531, GLU562, VAL492, LYS514, LEU484, ALA512, VAL492, LYS514, VAL561, and MET535
		1‐(3,6,6‐Trimethyl‐1,6,7,7a‐tetrahydrocyclopenta[c]pyran‐1‐yl) ethanone (PubChem ID: 605654)	−7.9	ASP641, VAL492, ILE545, VAL561, ALA512, LEU630, LYS514, MET535, VAL559, and VAL561
	1S1P	Diclofenac sodium (standard drug)	−8.5	ASN167, TYR24, HIS117, TYR55, and PHE306
		1‐(3,6,6‐Trimethyl‐1,6,7,7a‐tetrahydrocyclopenta[c]pyran‐1‐yl) ethanone	−8.8	GLN222, TYR24, TYR55, TRP227, PHE306, LEU54, and LYS270
Antiarthritic	3VP1	Indomethacin	−7.9	SER286, ASN335, TYR414, TYR249, TYR466, and VAL484
		4‐Hydroxy‐1H‐pyrazolo[3,4‐D] pyrimidine riboside	−7.3	PHE318, ASP467, LEU321, and SER314

Indomethacin, diclofenac sodium, and ascorbic acid were used as standard drugs across different targets. The compound 1‐(3,6,6‐trimethyl‐1,6,7,7a‐tetrahydrocyclopenta[c]pyran‐1‐yl) ethanone exhibited consistently strong binding across analgesic and anti‐inflammatory targets, suggesting its potential as a lead candidate. Similarly, 4‐hydroxy‐1H‐pyrazolo [3,4‐D] pyrimidine riboside showed potential antioxidant activity (binding affinity −8.6 kcal/mol, compared to −6.3 kcal/mol for its standard ascorbic acid) as well as analgesic and antiarthritic activity. The third component that has been picked up through docking studies is maltose, as it shows good interaction with one of the antioxidant targets. Previous studies have also reported that maltose has antioxidant activity better than sucrose and fructose. The antioxidant activity of maltose has been found to increase significantly when it is subjected to heat [62, 63].

Two proteins, PDB ID: 1DGH and 1XAN, were employed to evaluate antioxidant activity. With the protein PDB ID: 1DGH, the standard compound, ascorbic acid, showed moderate binding affinity (−6.5 kcal/mol) with contacts to ARG72 and SER114. In contrast, maltose exhibited a stronger binding affinity of −8.6 kcal/mol, interacting with residues like ILE332, TYR358, HIS362, and ARG365, suggesting potent antioxidant potential. Additionally, with the protein PDB ID: 1XAN, ascorbic acid again showed a modest affinity (−6.3 kcal/mol) with interactions to VAL329 and ASP331. The test ligand 4‐hydroxy‐1H‐pyrazolo [3,4‐D] pyrimidine riboside displayed a significantly enhanced binding (−8.6 kcal/mol), involving multiple polar and charged residues, including GLU50, ASP331, and THR156, indicating favorable binding within the antioxidant active site.

Table 8 demonstrates comparative pharmacokinetic properties of the three standard drugs, diclofenac (anti‐inflammatory), ascorbic acid (antioxidant), and indomethacin (NSAID), and three selected investigational compounds, 1‐(3,6,6‐trimethyl‐1,6,7,7a‐tetrahydrocyclopenta[c]pyran‐1‐yl) ethanone, 4‐hydroxy‐1H‐pyrazolo [3,4‐D] pyrimidine riboside, and maltose. For the calculation of the pharmacokinetic properties of the drugs under study with the target proteins, 1‐(3,6,6‐trimethyl‐1,6,7,7a‐tetrahydrocyclopenta[c]pyran‐1‐yl) ethanone, 4‐hydroxy‐1H‐pyrazolo [3,4‐D] pyrimidine riboside, and maltose, QikProp software was used.

**Table 8:** ADME properties of the ligand–protein complexes and standard drug.

Molecules	Absorption	Distribution			CNS permeability		
	%HOA	QPPCaco	QPPMDCK	QPlogKhsa	CNS	QPlogBB	PSA
Diclofenac sodium (standard drug)	100	345.796	758.298	0.059	−1	−0.213	57.889
Ascorbic acid (standard drug)	44.781	40.017	15.26	−0.94	−2	−1.755	127.371
Indomethacin (standard drug)	91.486	162.164	217.174	0.037	−1	−0.699	84.209
1‐(3,6,6‐Trimethyl‐1,6,7,7a‐tetrahydrocyclopenta[c]pyran‐1‐yl) ethanone (605654)	100	3648.454	2004.117	0.085	1	0.116	34.276
4‐Hydroxy‐1H‐pyrazolo[3,4‐D] pyrimidine riboside	48.466	55.935	21.915	−0.892	−2	−1.602	146.675
Maltose	0	7.777	2.598	−1.145	−2	−2.797	194.907

The pharmacokinetic properties of the standard drugs and selected ligands were analyzed using key pharmacokinetic descriptors, including human oral absorption (%HOA), permeability across Caco‐2 and MDCK cell lines (QPPCaco and QPPMDCK, respectively), plasma protein binding potential (QPlogKhsa), central nervous system (CNS) activity, blood–brain barrier permeability (QPlogBB), and polar surface area (PSA).

The standard drug diclofenac sodium (Table 8) exhibited excellent oral absorption (100% HOA), high membrane permeability (QPPCaco: 345.80; QPPMDCK: 758.30), moderate plasma protein binding (QPlogKhsa: 0.059), and limited CNS permeability (CNS: −1; QPlogBB: −0.213). Ascorbic acid, on the other hand, demonstrated lower oral absorption (44.78%) and poor membrane permeability (QPPCaco: 40.02; QPPMDCK: 15.26), likely due to its high polarity (PSA: 127.37), which is also reflected in its very low CNS permeability (QPlogBB: −1.755). Indomethacin showed favorable absorption (91.49%) and acceptable permeability, with moderate distribution parameters, though still exhibiting restricted CNS penetration.

Among the test ligands, 1‐(3,6,6‐trimethyl‐1,6,7,7a‐tetrahydrocyclopenta[c]pyran‐1‐yl) ethanone exhibited superior predicted pharmacokinetic characteristics, with excellent oral absorption (100%), extremely high permeability (QPPCaco: 3648.45; QPPMDCK: 2004.12), and positive CNS activity (CNS: 1; QPlogBB: 0.116). Its low PSA (34.28) suggests favorable membrane diffusion and BBB permeability. 4‐Hydroxy‐1H‐pyrazolo [3,4‐D] pyrimidine riboside presented moderate absorption (48.47%) with limited permeability and high polarity (PSA: 146.68), contributing to its poor CNS permeability. Maltose showed no oral absorption (0% HOA), negligible membrane permeability, and extremely high PSA (194.91), clearly indicating its poor drug‐likeness and limited potential for systemic or CNS‐related therapeutic applications.

For anti‐inflammatory assessment, two protein targets, 5UR1 and 1S1P, were evaluated. The standard drug diclofenac sodium demonstrated a binding affinity of −7.6 kcal/mol with 5UR1, forming interactions with key residues including GLU531, GLU562, LYS514, and VAL492. The test compound 1‐(3,6,6‐trimethyl‐1,6,7,7a‐tetrahydrocyclopenta[c]pyran‐1‐yl) ethanone exhibited a slightly improved binding affinity of −7.9 kcal/mol, engaging both polar and hydrophobic residues such as ILE545, MET535, VAL561, and ASP641, suggesting stronger inhibitory potential. Against 1S1P, diclofenac sodium showed a higher binding affinity of −8.5 kcal/mol, interacting with ASN167, HIS117, TYR55, and PHE306, while the test ligand surpassed this with a docking score of −8.8 kcal/mol, forming stable interactions with similar active site residues including TYR24, TRP227, and PHE306 and additional hydrogen bonding with GLN222, indicating enhanced binding stability and potential anti‐inflammatory activity.

Docking of ligands with cyclooxygenase (COX) enzymes (PDB IDs: 2OYE, 6COX, and 5C1M) revealed distinct variations in binding affinities and interaction profiles. For 2OYE, the standard drug indomethacin exhibited a strong binding affinity of −9.1 kcal/mol, forming key interactions with residues such as MET522, ILE523, SER353, TYR355, LEU93, VAL116, and LEU357, consistent with COX active site contacts. The test ligand 1‐(3,6,6‐trimethyl‐1,6,7,7a‐tetrahydrocyclopenta[c]pyran‐1‐yl) ethanone showed a slightly lower affinity (−7.4 kcal/mol) but maintained stable interactions with hydrophobic and polar residues, including ILE523, VAL349, ALA527, and TYR355. Similarly, 4‐hydroxy‐1H‐pyrazolo [3,4‐D] pyrimidine riboside demonstrated an identical docking score (−7.4 kcal/mol), forming hydrogen bonds with HIS388, TYR385, and ASN382. In the case of the crystal structure 6COX, indomethacin exhibited a lower affinity (−6.3 kcal/mol) with interactions involving THR94, HIS95, ASP515, and ALA516, whereas 1‐(3,6,6‐trimethyl‐…ethanone) showed enhanced binding (−8.0 kcal/mol), engaging residues such as ARG120, TYR355, LEU352, and ALA527, suggesting a stronger ligand–protein interaction. For the crystal structure 5C1M, indomethacin again showed favorable binding (−8.5 kcal/mol) through contacts with TYR148, ILE322, SER55, and TRP293, while 4‐hydroxy‐1H‐pyrazolo [3,4‐D] pyrimidine riboside had a slightly weaker interaction (−7.3 kcal/mol), primarily contacting TRP318, indicating reduced binding stability compared to the standard drug.

For antiarthritic potential, the target protein crystal structure 3VP1 was used. Indomethacin showed a docking score of −7.9 kcal/mol, forming hydrogen bonds and hydrophobic contacts with SER286, TYR414, TYR249, and VAL484. 4‐hydroxy‐1H‐pyrazolo [3,4‐D] pyrimidine riboside showed slightly reduced affinity (−7.3 kcal/mol) and interacted with residues including PHE318, ASP467, and SER314.

### 3.10. Molecular Dynamics Simulation

Molecular dynamics simulations are used to investigate the biomolecular behavior of the best two compounds with their respective protein complexes, providing insights into their alteration from their initial arrangement under physiological conditions [64]. Trajectory analysis encompassed RMSD, Rg, RMSF, SASA, and the total number of hydrogen bonds to assess the stability and dynamics of the complexes. MD simulation of the following compound–protein complex was analyzed: 1‐(3,6,6‐trimethyl‐1,6,7,7a‐tetrahydrocyclopenta[c]pyran‐1‐yl) ethanone with the protein 6COX (COX2_C18), 1S1P (1S1P_SL18), and 4‐hydroxy‐1H‐pyrazolo [3,4‐D] pyrimidine riboside with 1S1P (1XAN_C33). The detailed MD simulation data is provided in Supporting Information 3: File S3.

#### 3.10.1. RMSD

**Figure 6:**
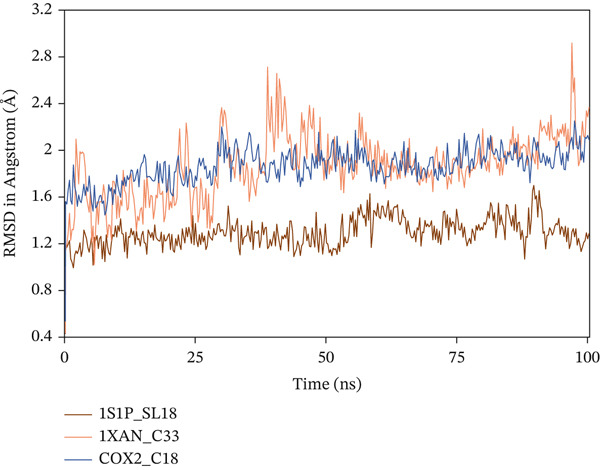
Analysis of the root mean square deviation (RMSD) of the C*α* atoms in the ligand–protein complexes throughout the 100 ns simulation.

The RMSD is a statistical method used to analyze the deviation of a molecular conformation from its initial state over a specific time period, providing information on the stability of the molecular system [65]. The study incorporated the C*α* atom′s deviation value to investigate the stability of the complexes, as demonstrated in Figure 6. The average RMSD values for 1S1P_SL18, 1XAN_C33, and COX2_C18 are 1.29, 1.859, and 1.86 Å, respectively, all of which are below 3.3 Å, signifying stability during the 100 ns simulation. The IXAN‐C33 showed abrupt increases at 39.5 and 96.75 ns, with deviation values of 2.7 and 2.9 Å, respectively, but these were within the standard limit. Overall, the complexes exhibited a consistent horizontal trend over the 100 ns simulation, demonstrating the stability of the protein–ligand interactions.

#### 3.10.2. RMSF

The RMSF serves as a quantitative approach to assess the dynamics of individual amino acids within the protein–ligand conformation, where the analysis reveals the residual vibration in 2FOM following ligand binding, offering valuable insights into the stability of the complex throughout the simulation duration [65]. Figure 7 shows the RMSF values derived from the single‐residue analysis for all complexes. Certain amino acid residues in different regions of the specific chains of all three proteins showed an unusual fluctuation. In the case of 1S1P_SL18, Residues 31, 133, 134, 136, and 320 had the highest RMSF peaks, with values ranging from 2.74 to 2.99 Å. Furthermore, Residues 462 to 478 in the 1XAN_C33 complex showed sudden increases in RMSF, spanning from 2.94 to 5.43 Å. In the COX2_C18 complex, 81A, 82A, 83A, 83B, 122B, and 278B residues of the A and B chains showed the most significant fluctuations, ranging from 3.05 to 3.39 Å. Thus, the homogeneity of the fluctuation trends for the 1S1P_SL18 and COX2_C18 complexes indicates their stability.

Figure 7Root mean square fluctuation (RMSF) analysis of the residues of the (a) 1S1P, (b) 1XAN, and (c) COX2 proteins upon compound binding over the 100 ns simulation.

**Figure:**
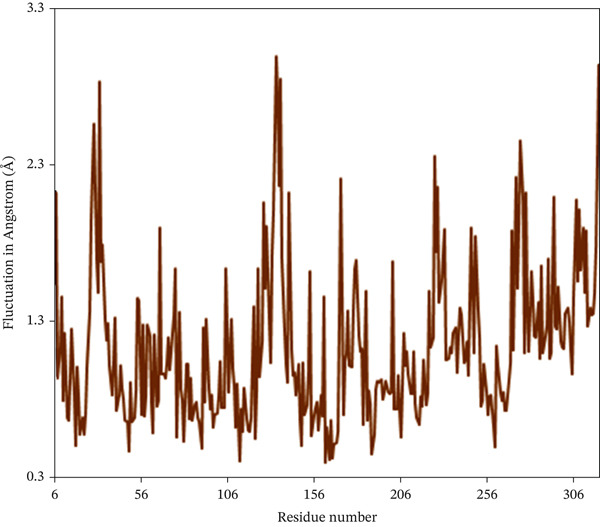
(a)

**Figure:**
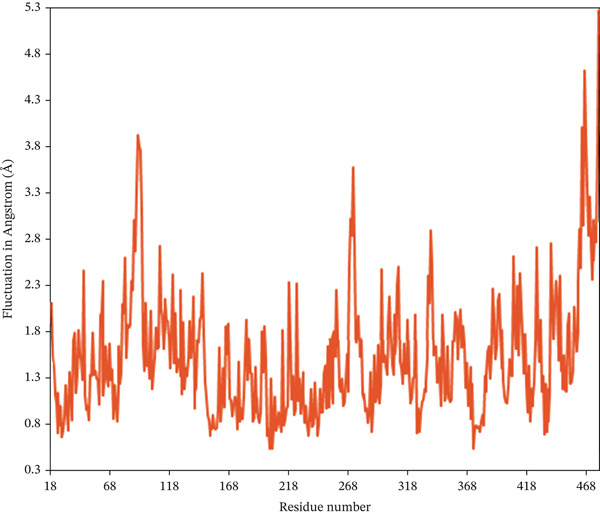
(b)

**Figure:**
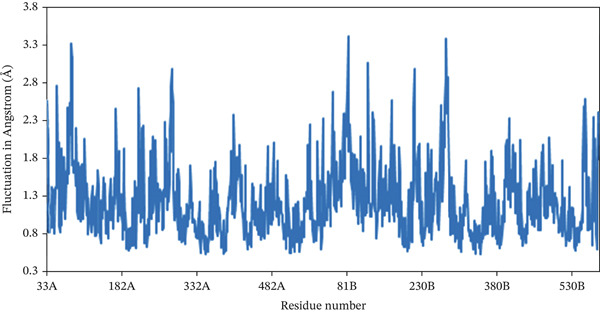
(c)

#### 3.10.3. Rg

The Rg analysis was conducted to evaluate the compactness and rigidity of the complex system, in which consistently lower fluctuations during a specific simulation period are associated with enhanced compactness and rigidity [66]. In the study, almost all the complexes exhibited a consistent fluctuation pattern throughout the 100 ns simulation, as shown in Figure 8. In 1S1P_SL18, the average Rg value was 19.284 Å, with a maximum deviation of 19.491 Å. With a range of 24.204–24.822 Å, which is also the maximum value, the Rg profile of 1XAN_C33 initially showed an inclining trend from 0 to 29.5 ns. But the value started to trend lower after 29.5 ns. The maximum Rg value for COX2_C18, measured at 33.75 ns, is 31.987 Å. Following that, there was a sharp drop from 33.75 to 66.75 ns. Nevertheless, this complex showed a steady variation trend beyond this time.

Figure 8Radius of gyration (Rg) analysis of the backbone structure of the ligand–protein complexes (a) 1S1P_SL18, (b) 1XAN_C33, and (c) COX2_C18 over the 100 ns simulation.

**Figure:**
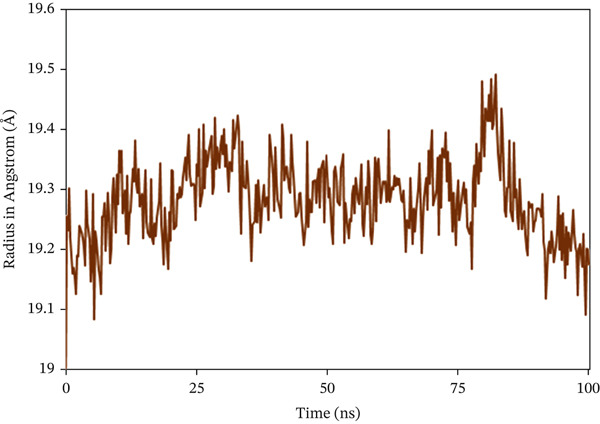
(a)

**Figure:**
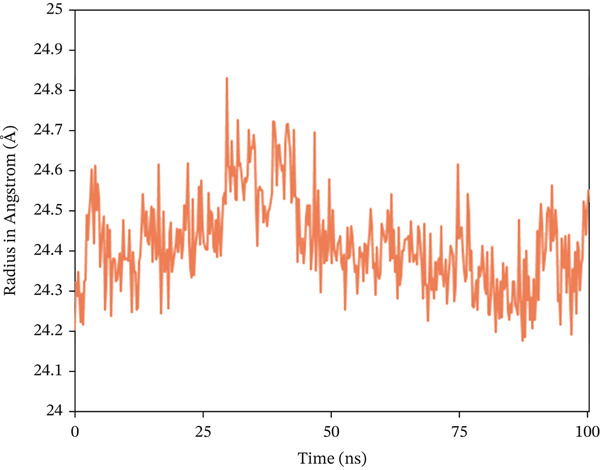
(b)

**Figure:**
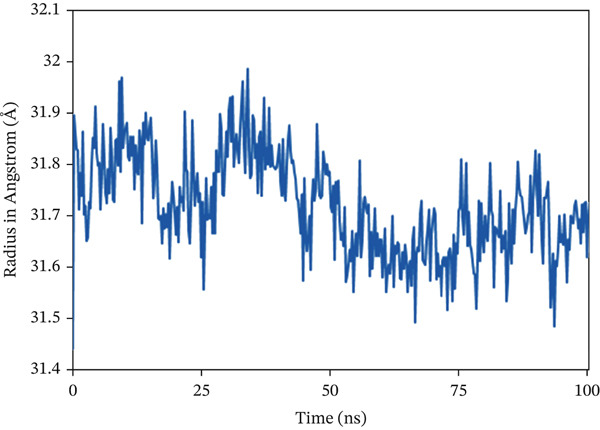
(c)

#### 3.10.4. SASA

The SASA analysis was utilized to investigate the increase in the complex′s surface area during the simulation, with higher SASA values indicating protein volume expansion, which appears to be unfavorable for the complex′s stability. Figure 9 shows that SASA values in nearly 1S1P_SL18, 1XAN_C33, and COX2_C18 complexes exhibited inconsistent fluctuation rates, with average surface areas of 13901.441, 21838.129, and 42775.049 Å^2^, respectively.

Figure 9Solvent‐accessible surface area (SASA) analysis of the structure of the (a) 1S1P_SL18, (b) 1XAN_C33, and (c) COX2_C18 complexes over the 100 ns simulation.

**Figure:**
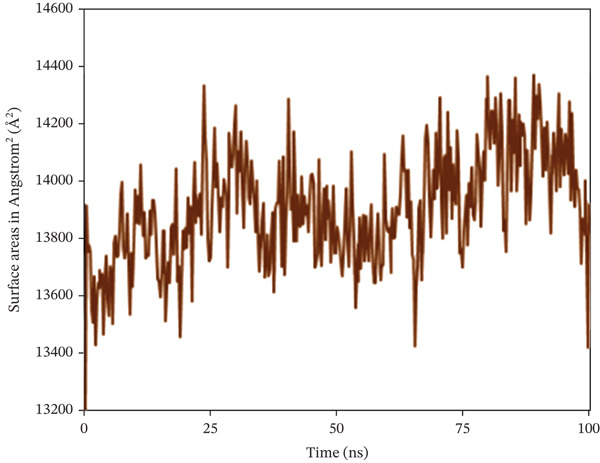
(a)

**Figure:**
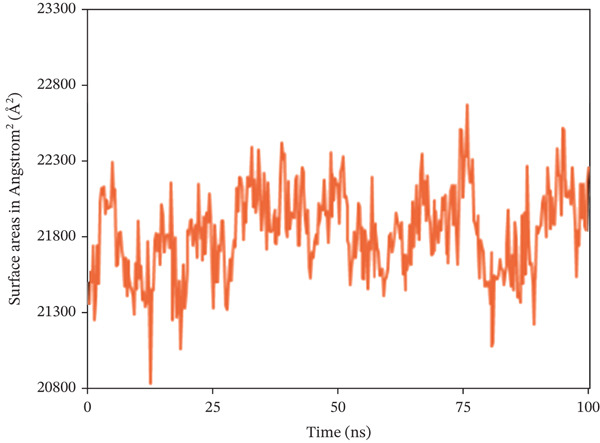
(b)

**Figure:**
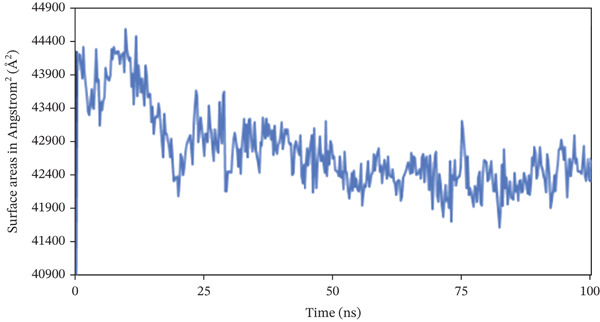
(c)

#### 3.10.5. Hydrogen Bonds

Hydrogen bonds not only significantly impact the stability of protein structures but also regulate various pharmacokinetic properties of a compound within a biological system. For this purpose, the study counted the total number of hydrogen bonds in the solute and those formed between the solute and the solvent during the 100 ns simulation period. Figure 10 shows the count of hydrogen bonds at specific nanosecond intervals. For 1S1P_SL18, the number of hydrogen bonds increased from 218 to 273, with an average of 239. Moreover, during a 100 ns simulation, hydrogen bonds in the 1XAN_C33 complex ranged from 313 to 368, with an average of 340. In the COX2_C18 complex, they ranged from 832 to 947, averaging 881. Thus, all the complexes demonstrated the ideal number of hydrogen bonds throughout the simulation.

Figure 10Illustrating the number of hydrogen bonds between solute and solvent extracted from (a) 1S1P_SL18, (b) 1XAN_C33, and (c) COX2_C18 complexes during a 100 ns simulation time.

**Figure:**
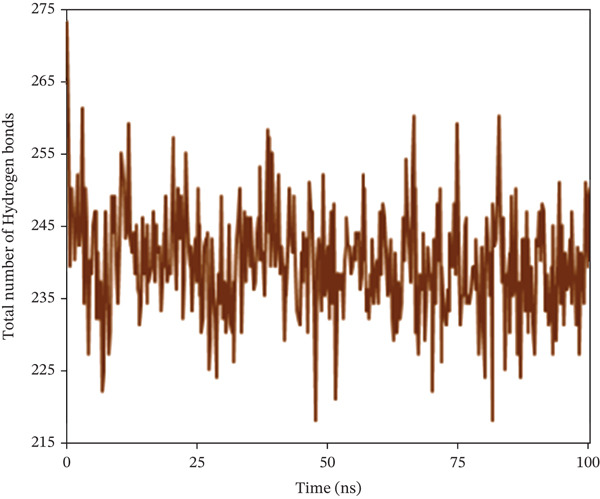
(a)

**Figure:**
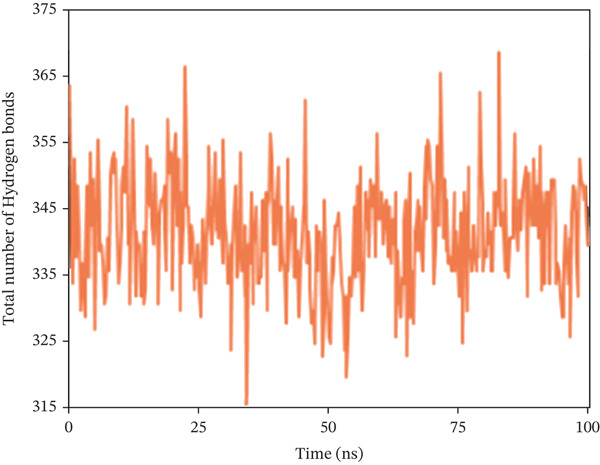
(b)

**Figure:**
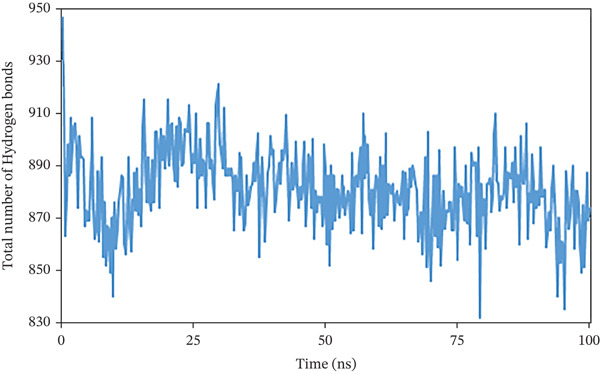
(c)

#### 3.10.6. Calculation of Binding Free Energy

The binding free energy was determined using the MMPBS method to assess the extent of interaction and conformational alterations within the drug–ligand complex following ligand binding. The result in Figure 11 shows a mean binding energy of 84.499, −193.949, and 74.362 kJ/mol, respectively, in 1S1P_SL18, 1XAN_C33, and COX2_C18 complexes. The positive value for the 1XAN_C33 complex suggested that ligand binding was nonspontaneous with this protein.

Figure 11The MM_PBSA values extracted from protein–ligand complexes, namely, (a) 1S1P_SL18, (b) 1XAN_C33, and (c) COX2_C18, concerning a 100 ns simulation time.

**Figure:**
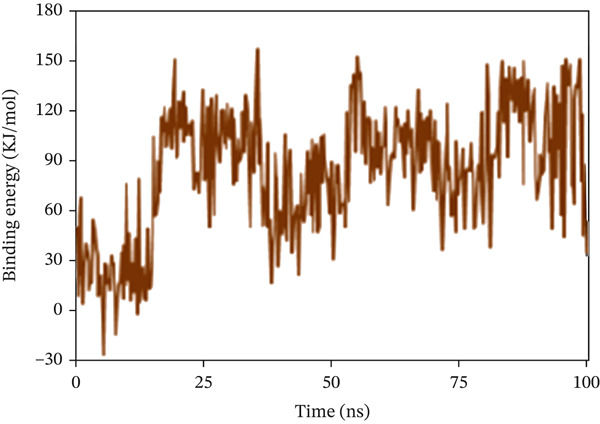
(a)

**Figure:**
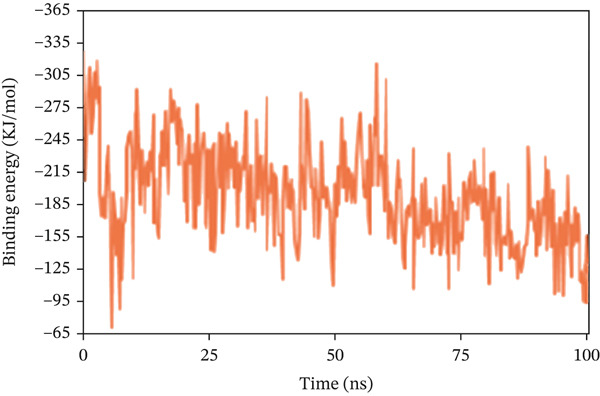
(b)

**Figure:**
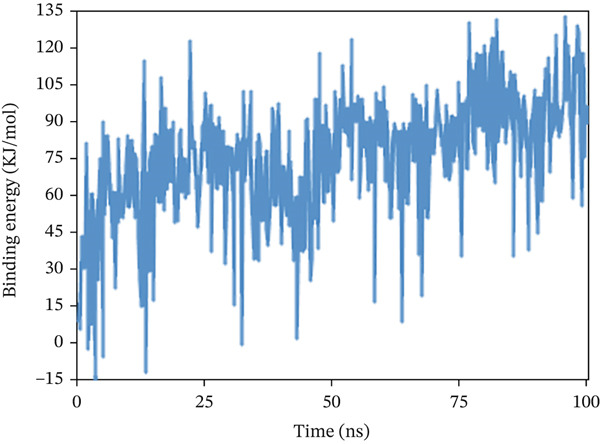
(c)

## 4. Discussion

Bioactive compounds derived from plants exhibit considerable potential as sources for various human therapeutics and play a significant role in global disease management. Their capacity to offer effective treatments with minimal side effects contributes to optimal safety [67–70]. Bangladesh contains a diverse range of medicinal plants. It is estimated that around 1000 of these are thought to have therapeutic properties, as indicated by traditional healers. The Ayurvedic, Unani, and homeopathic systems in this country have employed these natural substances to develop their medications [71, 72]. In Bangladesh, various parts of the *N. fruticans* plant have been employed in traditional medicine for a range of ailments, such as pain, diabetes, asthma, and tuberculosis, since ancient times [22, 28, 29]. While several pharmacological studies have examined nipa plum fruits, leaves, bark, and flowers, this study represents the first investigation into the antioxidant, analgesic, anti‐inflammatory, and antiarthritic properties of a methanolic extract of *N. fruticans* fruit husk. This was achieved through a combination of in silico methodologies, including molecular docking, amino acid interaction study, and ADME predictions. The application of in silico tools for assessing the toxicity of plant‐derived or synthetic compounds has become increasingly prevalent due to their efficiency in terms of cost and time. Following GC‐MS analysis, such computational approaches are frequently employed for the preliminary screening of phytochemicals. Among 82 compounds that were found through GC‐MS profiling, we selected 23 compounds for molecular docking as per their high retention time [73, 74].

The main reasons for measuring phenols, flavonoids, and tannins (collectively referred to as polyphenols) rather than other secondary metabolites are their high concentration in plant materials, their exceptional antioxidant capacity, and their robust, scientifically supported associations with both industrial and human health benefits [75]. Their main objective is to evaluate the antioxidant capacity of plant materials, which assist in addressing OS‐related conditions such as cancer, diabetes, and skin and cardiovascular disease, in addition to assessing their antibacterial, anti‐inflammatory, and therapeutic properties [76, 77]. Phenolic compounds derived from plants encompass phenolic acids, flavonoids, tannins, and the less prevalent stilbenes and lignans [78, 79]. Phenolic and flavonoid constituents are acknowledged as key natural components exhibiting antioxidant activity in plant‐based sources, which can mitigate the risk of various chronic diseases, including cancer, diabetes, and cardiovascular disease. Tannins can reduce lipid peroxidation and DNA mutations, while flavonoids exhibit antispasmodic, antiallergic, and anti‐inflammatory activities, along with protective effects on vascular and hepatic disorders [67–70, 80]. Comprehending the many functions of polyphenols enriches our understanding of plant‐derived substances and facilitates novel strategies in food preservation and the development of functional foods. As research progresses, the importance of these chemicals in enhancing health and well‐being becomes more apparent [75–77]. The TFC, TPC, and TTC of MNF extract were quantified in comparison to either gallic acid or quercetin through standard methodologies. According to the TFC, TPC, and TTC assays (Table 2), the MNF extract exhibited substantial levels of flavonoids and phenolic compounds, suggesting that the phenolic constituents of this plant are primarily glycosidic flavonoids. These glycosidic flavonoids are more soluble in polar solvents, which are more efficient than less polar solvents for extracting phenolic compounds from plant materials. Prior studies indicated that plants exhibiting elevated TPC demonstrated significant antioxidant properties [81]. Free radicals are typically generated as a result of normal metabolic processes in living organisms and are neutralized under healthy conditions by antioxidant molecules [82, 83]. The DPPH and FRAP assays are widely utilized methods for assessing the in vitro antioxidant activity of plant extracts [84, 85]. The capacity to scavenge the DPPH radical serves as an indicator of lipid peroxidation inhibition. Antioxidants neutralize the DPPH radical through the transfer of an electron or a hydrogen atom. This study demonstrated that the MNF extract exhibited comparable inhibitory effects on DPPH free radicals (IC_50_ 39.45 *μ*g/mL) to those of ascorbic acid (IC_50_ 36.28 *μ*g/mL). Prior studies demonstrated that the hydroxyl groups in phenolic compounds effectively trap free radicals. These compounds primarily function as reducing agents, hydrogen donors, singlet oxygen quenchers, or metal chelators [82, 86, 87]. The reducing ability test demonstrates that antioxidant compounds convert K_3_Fe(CN)_6_ (Fe^3+^) into potassium ferrocyanide, and the subsequent addition of FeCl_3_ results in the formation of Prussian blue. The data indicate significant DPPH scavenging activity (Table 3) and ferric reducing power assay results (Figure 1), underscoring the antioxidant potential of MNF extract.

The anti‐inflammatory effect of the MNF extract was examined using both in vitro (HRBC stabilization) and in vivo (carrageenan‐induced paw edema in rodents) methods. The HRBC membrane stabilization method was utilized, given that the membrane of RBC is analogous to the lysosomal membrane, and its stability indicates anti‐inflammatory properties [87, 88]. The stability of the lysosomal membrane inhibits inflammation and tissue damage by preventing the release of enzymes that induce inflammation [89]. This study demonstrates that the hemolysis of RBC is affected by the hypotonic nature of hyposaline, which induces cell membrane lysis, leading to the release of cellular fluids and electrolytes, ultimately resulting in cell shrinkage [88]. Five distinct concentrations (1000, 2000, 3000, 4000, and 5000 *μ*g/mL) of the extract were evaluated, revealing that the MNF extract demonstrated significant protection against hemolysis at 5000 *μ*g/mL (Figure 2), inhibiting the release of lytic enzymes and other inflammatory mediators in comparison to the standard indomethacin. The extract demonstrates membrane protective action due to its capacity to alter calcium concentration in the intercellular erythrocyte membrane [90]. The test results indicated significant inhibition of heat‐induced hemolysis (74.63*%* ± 0.84*%* at 5000 *μ*g/mL) by MNF extract, in comparison to the standard (80.88*%* ± 1.33*%* inhibition at 5000 *μ*g/mL). This in vitro study demonstrated a dose‐dependent inhibition (Table 5). The paw edema serves as an appropriate experimental model for assessing the antiedema effects of natural products. The in vivo evaluation demonstrated that the MNF extract exhibited a dose‐dependent inhibition of carrageenan‐induced inflammation symptoms, specifically the formation of hind paw edema, a recognized outcome of cellular inflammation [91]. Carrageenan‐induced paw edema is utilized to evaluate novel anti‐inflammatory drugs and to investigate the underlying mechanisms of inflammation. The initial phase (1 h) of this model entails the release of serotonin and histamine. The second phase, lasting over 1 h, is mediated by PGs and COX products. Edema is influenced by the interaction of kinin and polymorphonuclear leukocytes with proinflammatory factors such as PGs. MNF extract inhibits the biosynthetic pathway of PGs (E2 and D2) and protects animals from carrageenan‐induced lipid peroxidation by reducing NO radical production [91, 92]. The high levels of flavonoids, phenolics, saponins, and steroids in MNF extract may contribute to its strong anti‐inflammatory properties. The elevated TPC and TFC results of the extract suggest that its anti‐inflammatory effects may stem from the inhibition of COX1, which subsequently reduces the release of PGs and other mediators. The in vivo effect further supports the compound′s ability to mitigate inflammation in cells, with results demonstrating significance relative to other studies reporting similar dose‐dependent activities [91, 93].

Algesia, or pain, is consistently an unpleasant sensation. It is typically triggered by external or internal aversive stimuli. Recently, numerous detailed studies have been conducted regarding effective botanical species due to their beneficial effects on pain management. The peripheral and central analgesic activities of MNF were evaluated using the acetic acid–induced writhing test and the formalin‐induced paw licking test, respectively. In the writhing test, the injection of acetic acid stimulates the synthesis of PGs, leading to abdominal constrictions through the activation of peripheral nociceptors [80, 94]. The sensation of pain is modeled by the writhing response induced by acetic acid, which initiates a localized inflammatory response. It is hypothesized that peritoneal mast cells, acid‐sensing ion channels, and PG pathways mediate this response through the release of histamine, PGs, bradykinin, serotonin, COX, and cytokines [94]. NSAIDs, such as indomethacin, decrease PG levels, thereby diminishing nociceptor sensitivity to pain‐inducing agents like bradykinin [80, 95, 96]. The administration of MNF extract resulted in a significant (*p* < 0.0001) dose‐dependent reduction in the frequency of acetic acid–induced writhing (Figure 3). This observation indicates that the investigated extract exhibits peripherally mediated antinociceptive properties, potentially functioning through the reduction of PG synthesis or other inflammatory mediators. The formalin test was performed on the plant extracts to differentiate their central and peripheral antinociceptive activities. This method is acknowledged for generating distinct biphasic pain reactions, encompassing neurogenic and inflammatory phases, in response to various analgesics [94, 95]. The initial stage of pain perception, referred to as the neurogenic phase (0–5 min), involves the direct activation of nociceptors via the release of substance P. A*δ* fibers in the central nociceptive primary afferent terminals are responsible for detecting this stimulation. The late phase of pain perception, occurring during the inflammatory phase (15–30 min), is characterized by pain resulting from inflammation. This pain is mediated by inflammatory cytokines, including PGs, histamine, bradykinin, and serotonin in peripheral tissues. This late phase also involves the stimulation of spinal cord neurons located in the dorsal horns. Both phases possess distinct characteristics that can be employed to assess the antinociceptive effect and to clarify the mechanisms of antinociception. Our investigation revealed that the tested extract significantly (*p* < 0.01 and *p* < 0.05) decreased the mean time of paw licking in response to formalin injection during both phases (Table 6). Consequently, the results of our study indicate that MNF extract exhibits both peripheral and central antinociceptive effects, as well as additional anti‐inflammatory activity. Our tested extracts also contained bioactive phytoconstituents, including flavonoids, alkaloids, and terpenoids, which have been associated with significant analgesic and anti‐inflammatory effects in prior pharmacological studies [93–95].

Synthetic medications for RA exhibit specific limitations and adverse effects. Natural products are increasingly favored over conventional drugs due to their continuous availability, enhanced compatibility, cost‐effectiveness, reduced toxicity and side effects, greater safety, and improved efficacy. Protein denaturation is a primary manifestation of arthritic disease, potentially resulting in the formation of autoantigens under various conditions. Extrinsic stressors such as heat, organic solvents, strong acids, or bases can lead to the loss of secondary and tertiary protein structures, a phenomenon referred to as protein denaturation [35, 48]. Protein denaturation is implicated in inflammation, lipodystrophy, hyperlipidemia, vasomotor rhinitis, RA, atherosclerosis, cardiovascular diseases, cancer, kidney stones, and diabetes mellitus. Phytochemicals derived from plants that can inhibit protein denaturation may be beneficial for the development of antiarthritic, anti‐inflammatory, and analgesic medications [48, 96]. Electrostatic, hydrogen, hydrophobic, and disulfide bonding variations contribute to the denaturation mechanism [16, 49]. This study demonstrated that the MNF extract exhibited a dose‐dependent antiarthritic effect comparable to that of the reference medication, diclofenac sodium, in the in vitro protein denaturation model. The MNF extract exhibited the highest inhibition at a dosage of 500 *μ*g/mL, achieving 88.77*%* ± 0.31*%* (Figure 4). The observed increases in test sample absorbance relative to the control indicate that the tested extract has the potential to mitigate the heat denaturation of protein (albumin).

Toxicity evaluation is a crucial metric for understanding the hazardous index of the extract [38]. In this investigation, the mice in the experimental groups were administered the crude extract. The mice were observed daily until Day 14 for any indications of toxicity and mortality. The clinical symptom is a significant observation indicating the harmful effects on organs in the treated groups. In the 14‐day acute toxicity study, mice administered a single oral dose of 2000 mg/kg of methanolic seed extract exhibited no apparent signs of distress, and no obvious symptoms of toxicity or mortality were recorded. All the mice exhibited weight growth and showed no notable alterations in behavior. Additionally, physical characteristics, including skin, fur, and eyes, were observed to be normal. Although the body weight of the mice exhibited a rise, this suggests that the administration of the crude extract has a minor level of toxicity on the animals′ growth. Moreover, assessing food intake and H_2_O consumption is crucial in evaluating the safety of a therapeutic product, as adequate nutrient intake is vital for the animal′s physiological condition and the effective response to the tested medications. This study found that the administration of MNF extract did not influence food intake or H_2_O consumption, nor did it induce appetite suppression or cause any adverse consequences. Consequently, this signifies that there was no disruption in glucose, protein, or fat metabolism. The molecular docking study further supports the in vivo pharmacological activities. The stability of the ligand–protein interactions was confirmed by the MD simulation.

The MD simulations of the three protein–ligand complexes (1S1P_SL18, 1XAN_C33, and COX2_C18) over 100 ns show overall stability, with average C*α*‐RMSD values well below the 3.3 Å threshold, indicating maintained structural integrity throughout the trajectory. RMSF shows a few random fluctuations but a constant horizontal trend, suggesting stable binding, especially for 1S1P_SL18 and COX2_C18, whereas 1XAN_C33 exhibits higher fluctuations at specific residues without complete destabilization. The Rg results point to maintained compactness across complexes. However, transient trends occur in specific systems before stabilization, consistent with typical MD behavior under binding conditions, and SASA data show variable surface exposure without a universal destabilizing pattern, reflecting conformational adjustments during binding. Hydrogen bond networks remain substantial between solute and solvent and within complexes, supporting stability over the 100 ns window. MM‐PBSA binding energies indicate more favorable interactions for 1S1P_SL18 and COX2_C18, while 1XAN_C33 shows a less favorable, potentially nonspontaneous binding profile.

The primary objective of our investigation was to identify novel compounds from MNF extract that exhibit potential as analgesic, anti‐inflammatory, and antiarthritic agents with minimal side effects. The methanolic extract of *N. fruticans* husk has demonstrated antioxidant, analgesic, anti‐inflammatory, and antiarthritic properties through in vitro and in silico experiments. This resulted in the selection of certain phytoconstituents for further investigation. The standard problems and essential elements in the docking methodology encompass factors such as ligand and receptor conformation, flexibility, and cavity identification, among others [75]. These underscore the challenges and constraints inherent in the foundational theories. The plant requires additional validation in animal models to verify its antirheumatic efficacy in vivo, potentially leading to the development of a contemporary pharmaceutical derived from it.

## 5. Conclusions

The results of our investigation indicate that the methanolic extract of the fruit husk of *N. fruticans* has demonstrated considerable promise in the field of natural medicine. This extract is abundant in bioactive phytochemicals that have antioxidant, analgesic, anti‐inflammatory, and antiarthritic activities, which may be utilized to develop efficient treatments for various disorders. Within the four pharmacological categories, the plant extract had multitarget potential, surpassing or equaling reference medicines in anti‐inflammatory and antiarthritic efficacy and moderate analgesic activity, as shown by both in vitro and in vivo studies. Furthermore, the in silico study picked up three promising molecules that might be responsible for the mentioned pharmacological activities. The compound 1‐(3,6,6‐trimethyl‐1,6,7,7a‐tetrahydrocyclopenta[c]pyran‐1‐yl) ethanone showed comparable binding affinity for the protein drug targets for anti‐inflammatory and analgesic activities. Concurrently, maltose exhibited significant antioxidant properties. The molecule 4‐hydroxy‐1H‐pyrazolo [3,4‐D] pyrimidine riboside exhibited moderate yet consistent affinity across categories, signifying a wide, albeit less powerful, pharmacological profile. Molecular dynamics simulation of the drug–protein complex further shows the stability of the complexes over a period of 100 ns. Hence, these molecules can be used as promising starting points for further optimization in drug discovery for the treatment of pain and inflammation. In conclusion, the MNF extract has demonstrated considerable potential as a natural treatment for diverse health issues. Further investigation is essential to comprehensively ascertain the therapeutic potential of these substances and to formulate safe and effective treatment alternatives for patients.

**Nomenclature**
GC‐MS: gas chromatography–mass spectrometry
HRBC: human red blood cell
RBC: red blood cell
rpm: revolutions per minute
TPC: total phenolic content
TFC: total flavonoid content
TTC: total tannin content
DPPH: 2,2‐diphenyl‐1‐picrylhydrazyl
FRAP: ferric reducing antioxidant power
IC_50_
: half‐maximal inhibitory concentration
NO: nitric oxide

## Author Contributions

Conceptualization: F.A.R., N.Z.K., and Z.S. Methodology: F.A.R. Software: A.C.B., N.M., M.H.T., and I.J.B. Validation: A.P.A., N.Z.K., and A.R. Formal analysis: A.C.B., N.M., and M.H.T. Investigation: F.A.R. and A.C.B. Resources: F.A.R. Data curation: N.Z.K., A.C.B., N.M., and M.H.T. Writing—original draft preparation: F.A.R. and N.Z.K. Writing—review and editing: F.A.R., N.Z.K., Z.S., and I.J.B. Visualization and supervision: F.A.R. and N.Z.K.

## Funding

No funding was received for this manuscript.

## Conflicts of Interest

The authors declare no conflicts of interest.

## Supporting Information

Additional supporting information can be found online in the Supporting Information section.

## Supporting information


**Supporting Information 1** File S1: List of identified molecules by GC‐MS of the plant extract and molecular docking results of the selected molecules.

**Supporting Information 2** File S2: Results of biological tests.

**Supporting Information 3** File S3: Results of MD simulation study.

## Data Availability

The data that support the findings of this study are available in the Supporting Information of this article (Supporting Information 1, 2, and 3).
