# Supplementary material for: Antioxidant, Anti‐inflammatory, Analgesic, and Antiarthritic Potentialities of Methanolic Extract of Fruit Husk of Nypa fruticans Wurmb, Established by In Silico Analysis
Source: Biomed Res Int. 2026 Apr 15;2026:8861594. doi: 10.1155/bmri/8861594 (PMC13082471; doi:10.1155/bmri/8861594)

**Supplementary Table 1: Identified molecules by GCMS of the plant extract.**

| **SL NO** | **R. Time** | **Area%** | **Compound Name** | **Molecular formula** |
| --- | --- | --- | --- | --- |
| 1 | 3.52 | 0.42 | 1,4-Dioxan-2-ol | [C_4_H_8_O_3_](https://pubchem.ncbi.nlm.nih.gov/#query=C4H8O3) |
| 2 | 3.562 | 1.89 | Hydroxyacetic acid, hydrazide | [C_2_H_6_N_2_O_2_](https://pubchem.ncbi.nlm.nih.gov/#query=C2H6N2O2) |
| 3 | 3.626 | 1.88 | Propanoic acid, 2-oxo- | [C_3_H_4_O_3_](https://pubchem.ncbi.nlm.nih.gov/#query=C3H4O3) |
| 4 | 3.999 | 0.12 | Propanoic acid, 2-oxo-, methyl ester | [C_4_H_6_O_3_](https://pubchem.ncbi.nlm.nih.gov/#query=C4H6O3) |
| 5 | 4.166 | 0.05 | 2,2-Dimethoxybutane | [C_6_H_14_O_2_](https://pubchem.ncbi.nlm.nih.gov/#query=C6H14O2) |
| 6 | 5.019 | 0.12 | 3,3-Dimethoxy-2-butanone | [C_6_H_12_O_3_](https://pubchem.ncbi.nlm.nih.gov/#query=C6H12O3) |
| 7 | 5.101 | 0.11 | Butyl 2-(2-(2-methoxyethoxy)ethoxy)acetate | [C_11_H_22_O_5_](https://pubchem.ncbi.nlm.nih.gov/#query=C11H22O5) |
| 8 | 9.79 | 0.11 | Catechol | [C_6_H_6_O_2_](https://pubchem.ncbi.nlm.nih.gov/#query=C6H6O2) |
| 9 | 9.86 | 0.52 | 4-Vinylphenol | [C_8_H_8_O](https://pubchem.ncbi.nlm.nih.gov/#query=C8H8O) |
| 10 | 9.913 | 0.96 | 1H-Pyrrole-2,5-dione, 3-ethyl-4-methyl- | [C_7_H_9_NO_2_](https://pubchem.ncbi.nlm.nih.gov/#query=C7H9NO2) |
| 11 | 10.141 | 0.31 | 1H-Indene, 2,3-dihydro-1,1,5,6-tetramethyl- | [C_13_H_18_](https://pubchem.ncbi.nlm.nih.gov/#query=C13H18) |
| 12 | 10.716 | 5.39 | 2-Methoxy-4-vinylphenol | [C_9_H_10_O_2_](https://pubchem.ncbi.nlm.nih.gov/#query=C9H10O2) |
| 13 | 10.985 | 0.11 | 4-Methyl-4-phenyl-2,3:5,6-diepoxycyclohexanone | [C_13_H_12_O_3_](https://pubchem.ncbi.nlm.nih.gov/#query=C13H12O3) |
| 14 | 11.136 | 0.08 | Phenol, 2,6-dimethoxy- | [C_8_H_10_O_3_](https://pubchem.ncbi.nlm.nih.gov/#query=C8H10O3) |
| 15 | 11.234 | 0.31 | 2-Propanone, 1-(3,5,5-trimethyl-2-cyclohexen-1-ylidene)-, (Z)- | [C_12_H_18_O](https://pubchem.ncbi.nlm.nih.gov/#query=C12H18O) |
| 16 | 11.27 | 0.07 | 12,15-Octadecadiynoic acid, methyl ester | [C_19_H_30_O_2_](https://pubchem.ncbi.nlm.nih.gov/#query=C19H30O2) |
| 17 | 11.442 | 0.5 | 2,6,6-Trimethylcyclohexa-1,4-dienecarbaldehyde | [C_10_H_14_O](https://pubchem.ncbi.nlm.nih.gov/#query=C10H14O) |
| 18 | 11.519 | 1.49 | 1-(3,6,6-Trimethyl-1,6,7,7a-tetrahydrocyclopenta[c]pyran-1-yl)ethanone | [C_13_H_18_O_2_](https://pubchem.ncbi.nlm.nih.gov/#query=C13H18O2) |
| 19 | 11.615 | 0.25 | (3S,4R,5R,6R)-4,5-Bis(hydroxymethyl)-3,6-dimethylcyclohexene | [C_10_H_18_O_2_](https://pubchem.ncbi.nlm.nih.gov/#query=C10H18O2) |
| 20 | 11.789 | 0.4 | Cyclohexene, 1-formyl-2-phenylsulfinylmethyl-3,3-dimethyl- | [C_16_H_20_O_2_S](https://pubchem.ncbi.nlm.nih.gov/#query=C16H20O2S) |
| 21 | 11.886 | 2.47 | Benzeneacetic acid, 4-methoxy-, methyl ester | [C_10_H_12_O_3_](https://pubchem.ncbi.nlm.nih.gov/#query=C10H12O3) |
| 22 | 12.12 | 0.47 | 3-(4-Allyl-2-methoxyphenoxy)-1,2-propanediol | [C_13_H_18_O_4_](https://pubchem.ncbi.nlm.nih.gov/#query=C13H18O4) |
| 23 | 12.225 | 0.32 | Formic acid, decyl ester | [C_11_H_22_O_2_](https://pubchem.ncbi.nlm.nih.gov/#query=C11H22O2) |
| 24 | 12.299 | 0.55 | 4-(2,6,6-Trimethylcyclohexa-1,3-dienyl)but-3-en-2-one | [C_13_H_18_O](https://pubchem.ncbi.nlm.nih.gov/#query=C13H18O) |
| 25 | 12.514 | 5 | Benzeneacetic acid, 3-methoxy- | [C_9_H_10_O_3_](https://pubchem.ncbi.nlm.nih.gov/#query=C9H10O3) |
| 26 | 12.7 | 0.3 | 5-Methoxy-3,4,4,6-tetramethyl-7-oxa-bicyclo[4.1.0]heptan-2-one | [C_11_H_18_O_3_](https://pubchem.ncbi.nlm.nih.gov/#query=C11H18O3) |
| 27 | 12.832 | 2.7 | DL-Arabinitol | [C_5_H_12_O_5_](https://pubchem.ncbi.nlm.nih.gov/#query=C5H12O5) |
| 28 | 12.928 | 1.45 | 2(4H)-Benzofuranone, 5,6,7,7a-tetrahydro-4,4,7a-trimethyl-, (R)- | [C_11_H_16_O_2_](https://pubchem.ncbi.nlm.nih.gov/#query=C11H16O2) |
| 29 | 13.061 | 3.5 | Phenol, 4-ethenyl-2,6-dimethoxy- | [C_10_H_12_O_3_](https://pubchem.ncbi.nlm.nih.gov/#query=C10H12O3) |
| 30 | 13.273 | 1.67 | .alpha.-D-Glucopyranoside, methyl | [C_7_H_14_O_6_](https://pubchem.ncbi.nlm.nih.gov/#query=C7H14O6) |
| 31 | 13.402 | 0.43 | Ethyl iso-allocholate | [C_26_H_44_O_5_](https://pubchem.ncbi.nlm.nih.gov/#query=C26H44O5) |
| 32 | 13.54 | 0.8 | Bicyclo[2.2.1]heptan-2-one, 1-(bromomethyl)-7,7-dimethyl-, (1S)- | [C_10_H_15_BrO](https://pubchem.ncbi.nlm.nih.gov/#query=C10H15BrO) |
| 33 | 13.578 | 1.42 | 4-Hydroxy-1H-pyrazolo[3,4-D]pyrimidine riboside | [C_10_H_12_N_4_O_5_](https://pubchem.ncbi.nlm.nih.gov/#query=C10H12N4O5) |
| 34 | 13.7 | 0.38 | 6-(3-Hydroxy-but-1-enyl)-1,5,5-trimethyl-7-oxabicyclo[4.1.0]heptan-2-ol | [C_13_H_22_O_3_](https://pubchem.ncbi.nlm.nih.gov/#query=C13H22O3) |
| 35 | 13.77 | 0.9 | Betamethasone valerate | [C_27_H_37_FO_6_](https://pubchem.ncbi.nlm.nih.gov/#query=C27H37FO6) |
| 36 | 13.955 | 1.23 | Maltose | [C_12_H_22_O_11_](https://pubchem.ncbi.nlm.nih.gov/#query=C12H22O11) |
| 37 | 14.066 | 0.75 | 2-Dodecen-1-yl(-)succinic anhydride | [C_16_H_26_O_3_](https://pubchem.ncbi.nlm.nih.gov/#query=C16H26O3) |
| 38 | 14.195 | 0.72 | Tetrahydroedulan | [C_13_H_24_O](https://pubchem.ncbi.nlm.nih.gov/#query=C13H24O) |
| 39 | 14.255 | 0.2 | 2-Butanone, 4-(2,2,6-trimethylcyclohexyl)- | [C_13_H_24_O](https://pubchem.ncbi.nlm.nih.gov/#query=C13H24O) |
| 40 | 14.305 | 0.25 | 4-(1-Hydroxyallyl)-2-methoxyphenol | [C_10_H_12_O_3_](https://pubchem.ncbi.nlm.nih.gov/#query=C10H12O3) |
| 41 | 14.401 | 0.51 | 2-(tert-Pentyl)cyclohexyl acetate | [C_13_H_24_O_2_](https://pubchem.ncbi.nlm.nih.gov/#query=C13H24O2) |
| 42 | 14.469 | 4.55 | (-)-Camphanic acid | [C_10_H_14_O_4_](https://pubchem.ncbi.nlm.nih.gov/#query=C10H14O4) |
| 43 | 14.654 | 0.5 | Ethanol, 2-(hexadecyloxy)- | [C_18_H_38_O_2_](https://pubchem.ncbi.nlm.nih.gov/#query=C18H38O2) |
| 44 | 14.937 | 0.1 | 2-Propanone, 1-(4-hydroxy-3-methoxyphenyl)- | [C_10_H_12_O_3_](https://pubchem.ncbi.nlm.nih.gov/#query=C10H12O3) |
| 45 | 15.089 | 5.27 | (E)-4-(3-Hydroxyprop-1-en-1-yl)-2-methoxyphenol | [C_10_H_12_O_3_](https://pubchem.ncbi.nlm.nih.gov/#query=C10H12O3) |
| 46 | 15.326 | 0.92 | 4-Oxo-.beta.-isodamascol | [C_13_H_20_O_2_](https://pubchem.ncbi.nlm.nih.gov/#query=C13H20O2) |
| 47 | 15.591 | 1.96 | Loliolide | [C_11_H_16_O_3_](https://pubchem.ncbi.nlm.nih.gov/#query=C11H16O3) |
| 48 | 15.75 | 0.19 | (3aR,5aS,9aS,9bR)-3a,6,6,9a-Tetramethyldecahydronaphtho[2,1-b]furan-2(1H)-one | [C_16_H_26_O_2_](https://pubchem.ncbi.nlm.nih.gov/#query=C16H26O2) |
| 49 | 16.176 | 0.44 | Neophytadiene | [C_20_H_38_](https://pubchem.ncbi.nlm.nih.gov/#query=C20H38) |
| 50 | 16.275 | 0.14 | 2-Pentadecanone, 6,10,14-trimethyl- | [C_18_H_36_O](https://pubchem.ncbi.nlm.nih.gov/#query=C18H36O) |
| 51 | 16.355 | 0.19 | 4,11-Dimethyl-8-(propan-2-yl)-5,12-dioxatricyclo[9.1.0.04,6]dodecan-7-ol, Me | **Not found in PubChem** |
| 52 | 16.439 | 0.28 | 2-Propenoic acid, 3-(4-hydroxy-3-methoxyphenyl)-, methyl ester | [C_11_H_12_O_4_](https://pubchem.ncbi.nlm.nih.gov/#query=C11H12O4) |
| 53 | 16.525 | 0.08 | 3,7,11,15-Tetramethyl-2-hexadecen-1-ol | [C_20_H_40_O](https://pubchem.ncbi.nlm.nih.gov/#query=C20H40O) |
| 54 | 17.395 | 0.67 | 6-Octadecenoic acid, methyl ester, (Z)- | [C_19_H_36_O_2_](https://pubchem.ncbi.nlm.nih.gov/#query=C19H36O2) |
| 55 | 17.471 | 4.73 | Hexadecanoic acid, methyl ester | [C_17_H_34_O_2_](https://pubchem.ncbi.nlm.nih.gov/#query=C17H34O2) |
| 56 | 18.01 | 3.18 | l-(+)-Ascorbic acid 2,6-dihexadecanoate | [C_38_H_68_O_8_](https://pubchem.ncbi.nlm.nih.gov/#query=C38H68O8) |
| 57 | 18.255 | 0.3 | Tetradecanamide | [C_14_H_29_NO](https://pubchem.ncbi.nlm.nih.gov/#query=C14H29NO) |
| 58 | 18.524 | 0.37 | Hexadecanoic acid, ethyl ester | [C_18_H_36_O_2_](https://pubchem.ncbi.nlm.nih.gov/#query=C18H36O2) |
| 59 | 18.636 | 2.72 | trans-Sinapyl alcohol | [C_11_H_14_O_4_](https://pubchem.ncbi.nlm.nih.gov/#query=C11H14O4) |
| 60 | 19.047 | 0.07 | Heptadecanoic acid, methyl ester | [C_18_H_36_O_2_](https://pubchem.ncbi.nlm.nih.gov/#query=C18H36O2) |
| 61 | 20.171 | 0.86 | 9,11-Octadecadienoic acid, methyl ester, (E,E)- | [C_19_H_34_O_2_](https://pubchem.ncbi.nlm.nih.gov/#query=C19H34O2) |
| 62 | 20.275 | 1.96 | 8,11,14-Docosatrienoic acid, methyl ester | [C_23_H_40_O_2_](https://pubchem.ncbi.nlm.nih.gov/#query=C23H40O2) |
| 63 | 20.428 | 1.57 | Phytol | [C_20_H_40_O](https://pubchem.ncbi.nlm.nih.gov/#query=C20H40O) |
| 64 | 20.694 | 1.28 | Methyl stearate | [C_19_H_38_O_2_](https://pubchem.ncbi.nlm.nih.gov/#query=C19H38O2) |
| 65 | 20.852 | 0.48 | Trichothec-9-en-8-one, 12,13-epoxy-4-hydroxy-, (4.beta.)- | [C_15_H_20_O_4_](https://pubchem.ncbi.nlm.nih.gov/#query=C15H20O4) |
| 66 | 20.916 | 0.43 | 7-Tetradecenal, (Z)- | [C_14_H_26_O](https://pubchem.ncbi.nlm.nih.gov/#query=C14H26O) |
| 67 | 21.295 | 0.39 | Octadecanoic acid | [C_18_H_36_O_2_](https://pubchem.ncbi.nlm.nih.gov/#query=C18H36O2) |
| 68 | 21.385 | 0.15 | 9,12,15-Octadecatrienoic acid, ethyl ester, (Z,Z,Z)- | [C_20_H_34_O_2_](https://pubchem.ncbi.nlm.nih.gov/#query=C20H34O2) |
| 69 | 21.613 | 1.46 | Hexadecanamide | [C_16_H_33_NO](https://pubchem.ncbi.nlm.nih.gov/#query=C16H33NO) |
| 70 | 22.128 | 0.29 | 2-Hexadecen-1-ol, 3,7,11,15-tetramethyl-, acetate, [R-[R*,R*-(E)]]- | [C_22_H_42_O_2_](https://pubchem.ncbi.nlm.nih.gov/#query=C22H42O2) |
| 71 | 22.772 | 0.24 | Undec-10-ynoic acid, tridec-2-yn-1-yl ester | [C_24_H_40_O_2_](https://pubchem.ncbi.nlm.nih.gov/#query=C24H40O2) |
| 72 | 22.914 | 0.12 | 9-Octadecenoic acid (Z)-, phenylmethyl ester | [C_25_H_40_O_2_](https://pubchem.ncbi.nlm.nih.gov/#query=C25H40O2) |
| 73 | 23.57 | 0.19 | 1,6,10,14-Phytatetraene-3,5,9-triol, trimethyl ether | **Not found in PubChem** |
| 74 | 23.703 | 0.28 | Epoxylathyrol | [C_20_H_30_O_5_](https://pubchem.ncbi.nlm.nih.gov/#query=C20H30O5) |
| 75 | 23.875 | 0.11 | .alpha.,.beta.-D-Glucopyranoside, 1-deoxy-1-undecylthio- | [C_17_H_34_O_5_S](https://pubchem.ncbi.nlm.nih.gov/#query=C17H34O5S) |
| 76 | 24.535 | 0.82 | 9,12-Octadecadienoic acid (Z,Z)-, 2,3-dihydroxypropyl ester | [C_21_H_38_O_4_](https://pubchem.ncbi.nlm.nih.gov/#query=C21H38O4) |
| 77 | 24.631 | 13.6 | Palmitoleamide | [C_16_H_31_NO](https://pubchem.ncbi.nlm.nih.gov/#query=C16H31NO) |
| 78 | 25.063 | 0.98 | Octadecanamide | [C_18_H_37_NO](https://pubchem.ncbi.nlm.nih.gov/#query=C18H37NO) |
| 79 | 25.666 | 0.13 | Ingol 12-acetate | [C_22_H_32_O_7_](https://pubchem.ncbi.nlm.nih.gov/#query=C22H32O7) |
| 80 | 26.81 | 0.07 | Pentadecafluorooctanoic acid, undec-2-en-1-yl ester | [C_19_H_21_F_15_O_2_](https://pubchem.ncbi.nlm.nih.gov/#query=C19H21F15O2) |
| 81 | 27.074 | 0.42 | Hexadecanoic acid, 2-hydroxy-1-(hydroxymethyl)ethyl ester | [C_19_H_38_O_4_](https://pubchem.ncbi.nlm.nih.gov/#query=C19H38O4) |
| 82 | 39.47 | 0.42 | .gamma.-Sitosterol | [C_29_H_50_O](https://pubchem.ncbi.nlm.nih.gov/#query=C29H50O) |

| **Table 2: 3D and 2D Interactions of the Ligand-Protein Complexes** | | | |
| --- | --- | --- | --- |
| **Protein [PDB]** | **Ligand** | **3D interactions** | **2D interactions** |
| COX1  [2OYE] | 1-(3,6,6-Trimethyl-1,6,7,7a-tetrahydrocyclopenta[c]pyran-1-yl)ethanone | 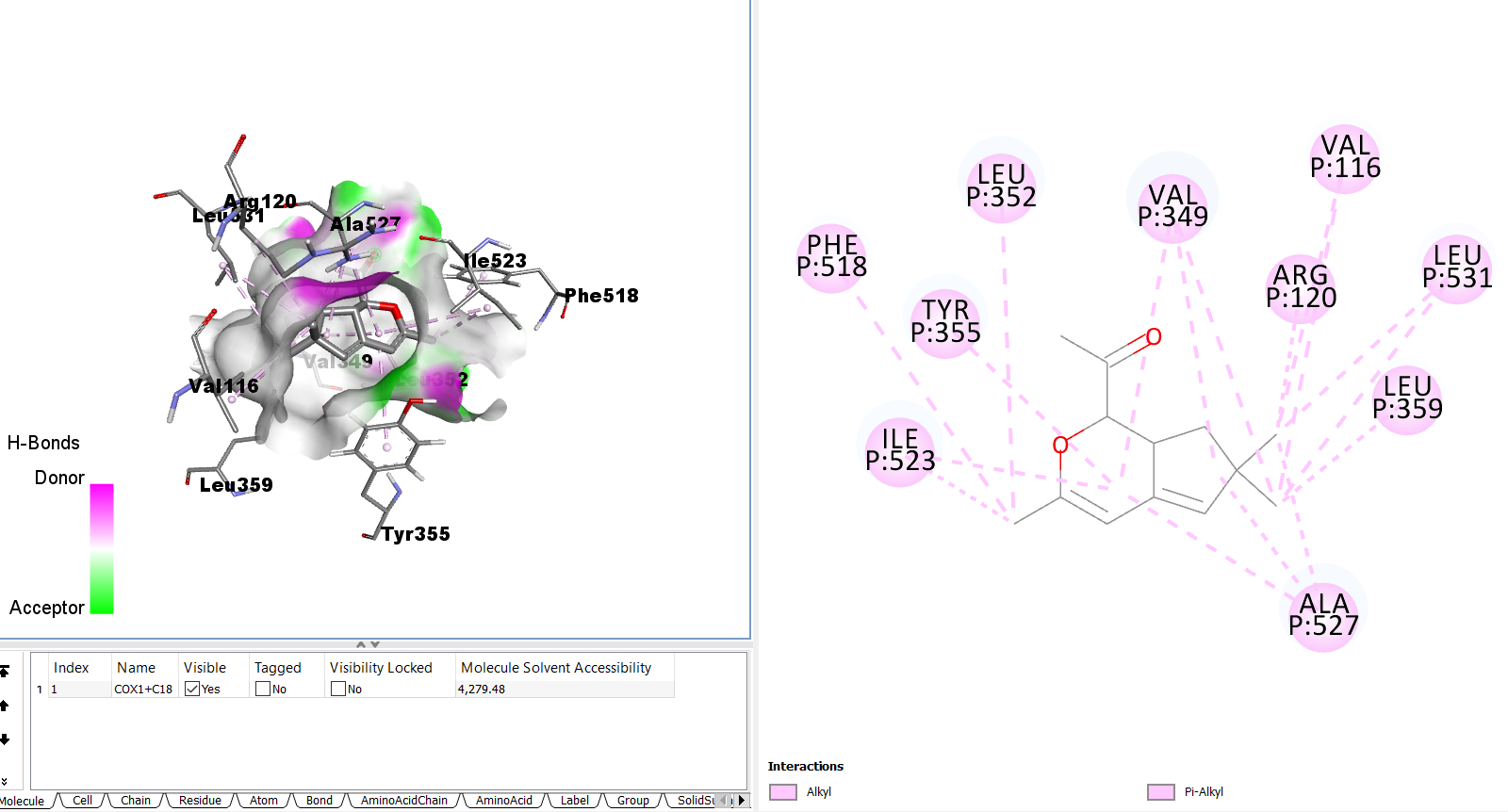 | 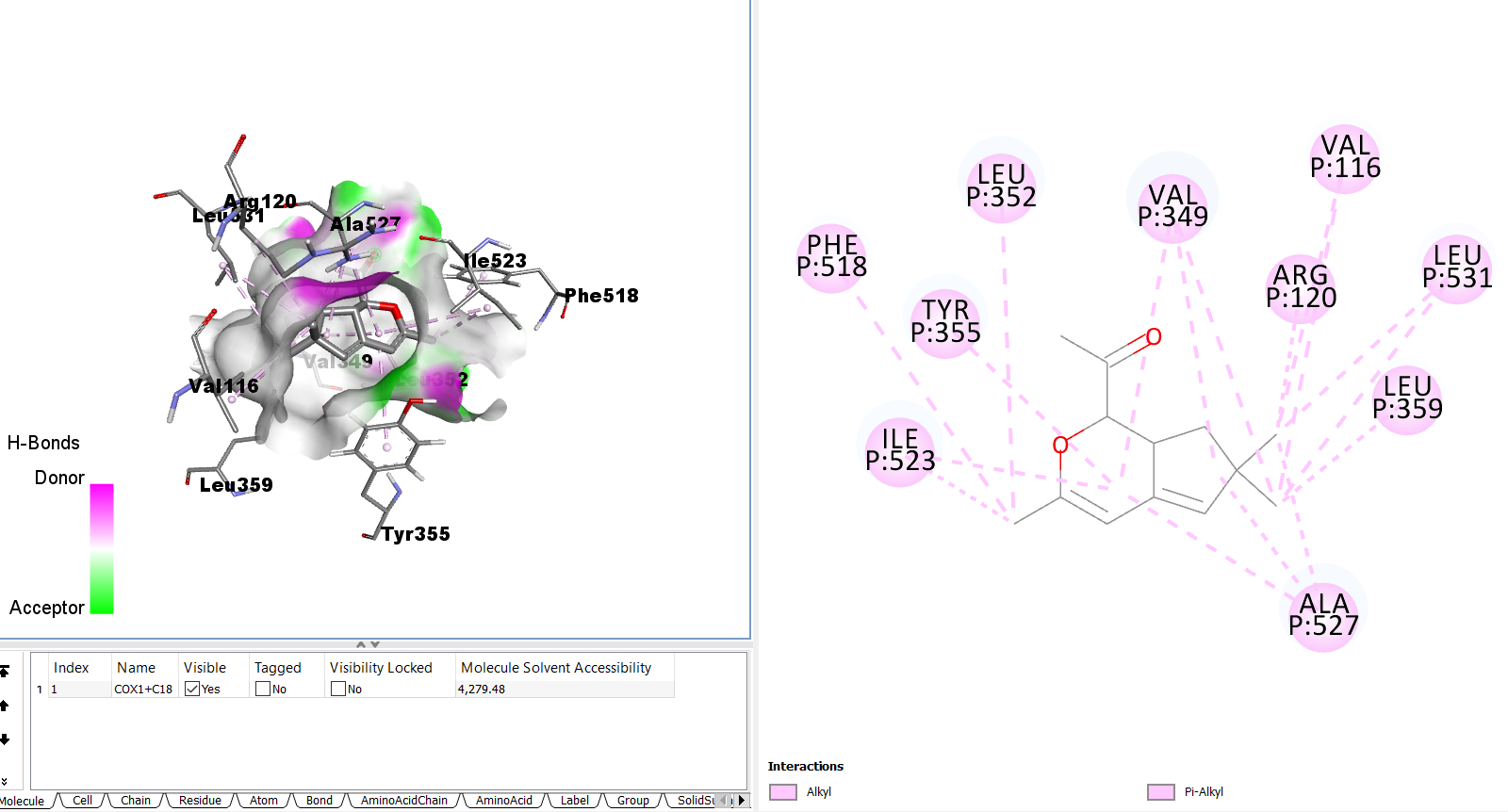 |
|  | 4-Hydroxy-1H-pyrazolo[3,4-D]pyrimidine riboside | 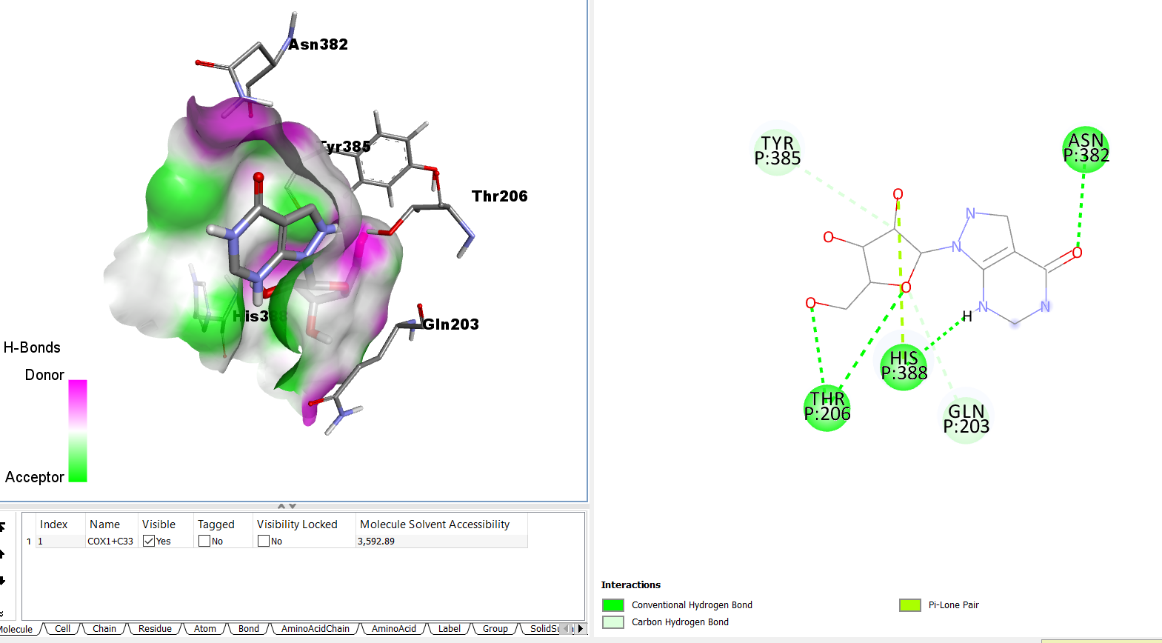 | 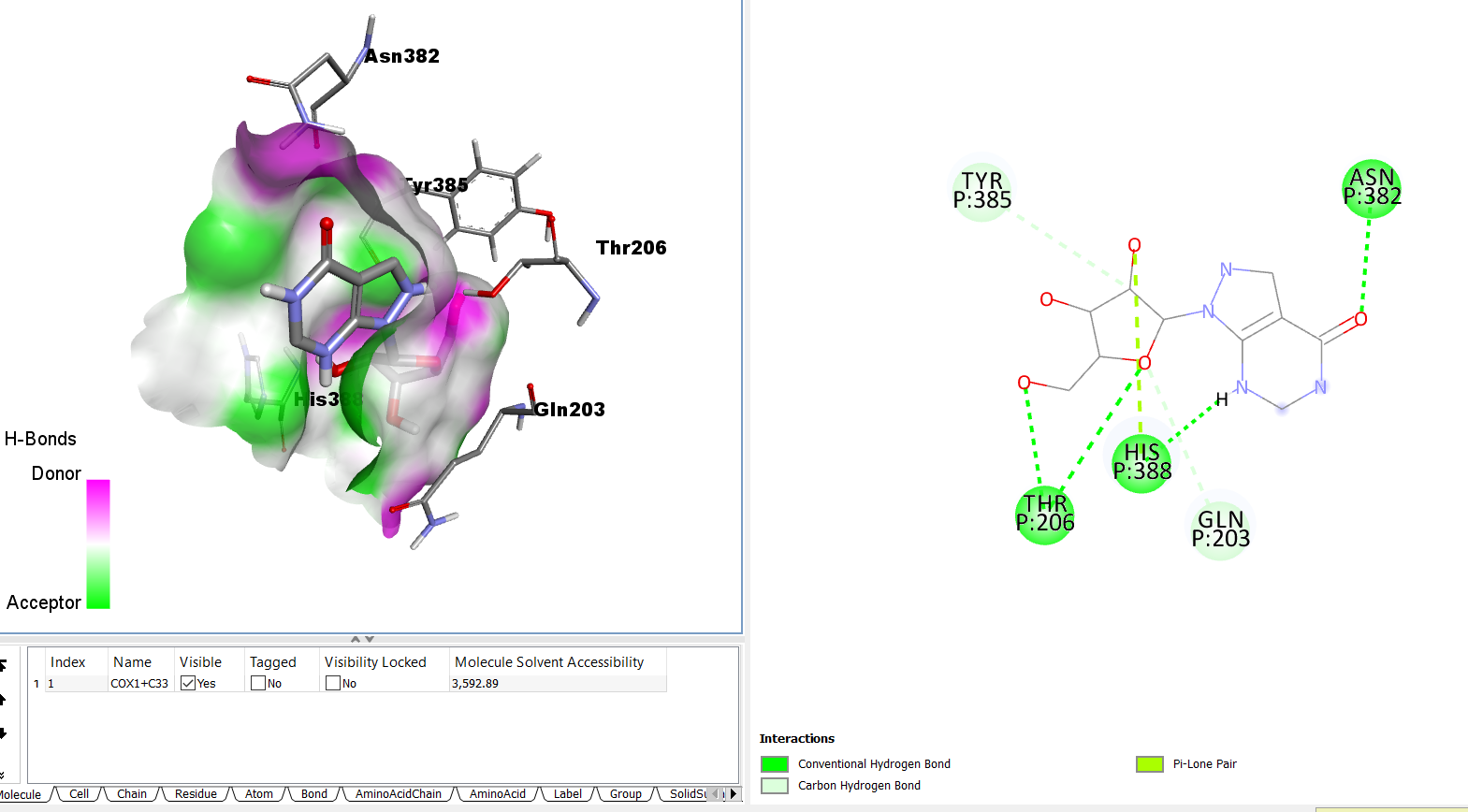 |
| COX2  [6COX] | 1-(3,6,6-Trimethyl-1,6,7,7a-tetrahydrocyclopenta[c]pyran-1-yl)ethanone | 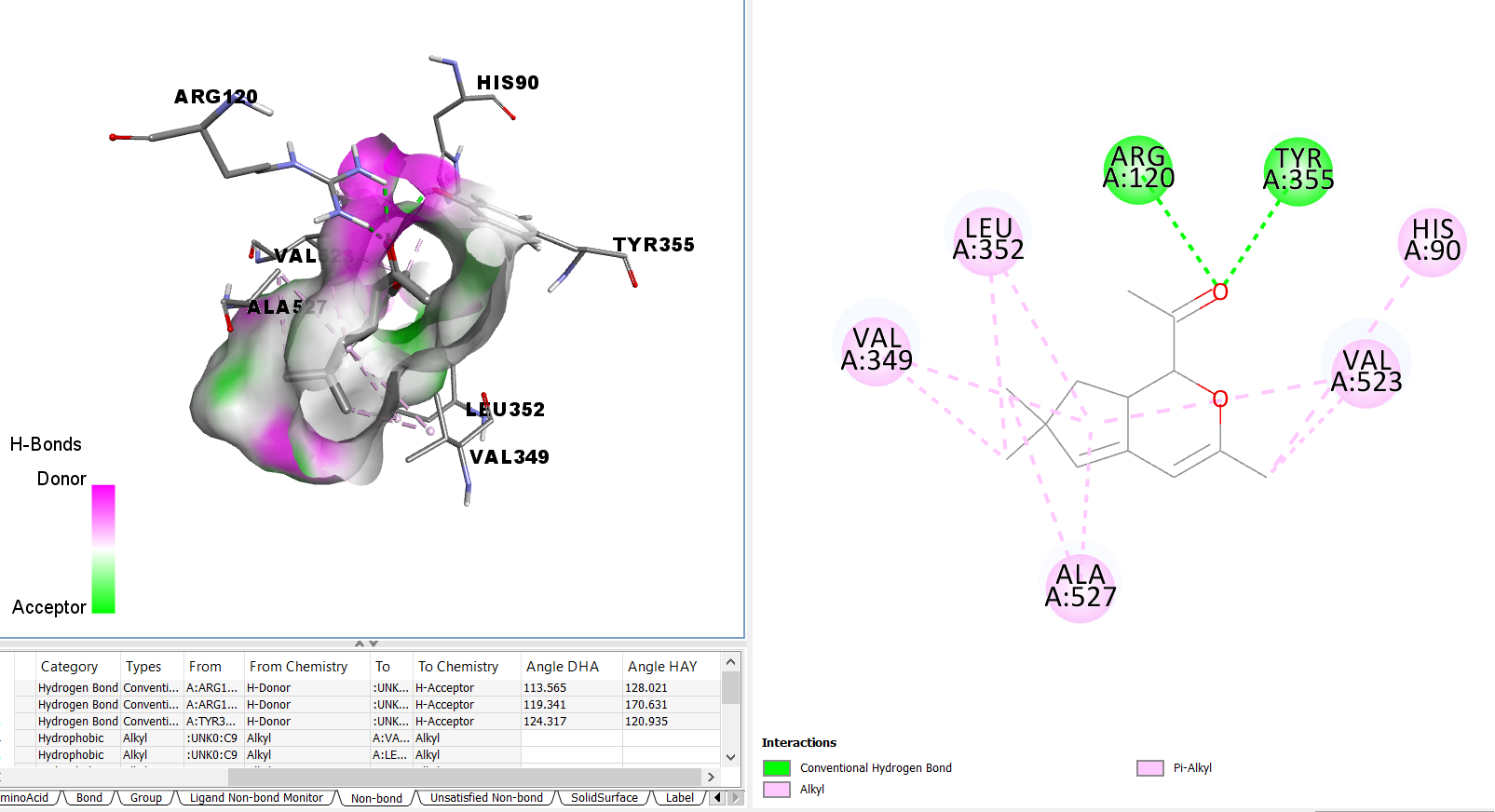 | 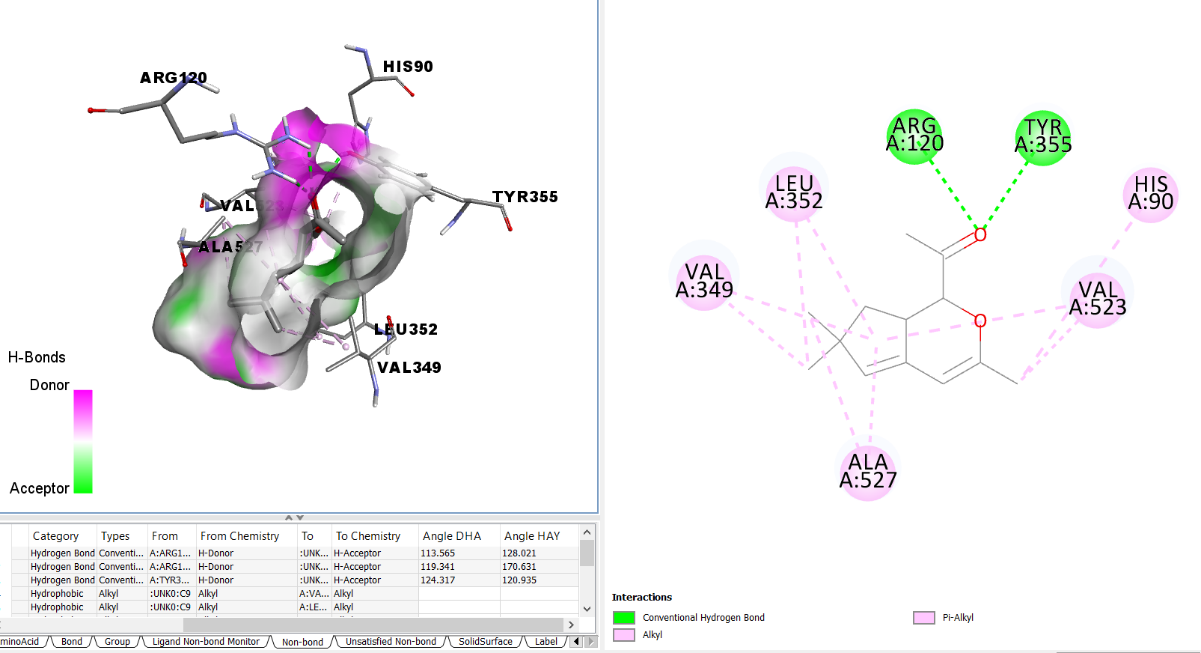 |
| Mu  [5C1M] | 4-Hydroxy-1H-pyrazolo[3,4-D]pyrimidine riboside | 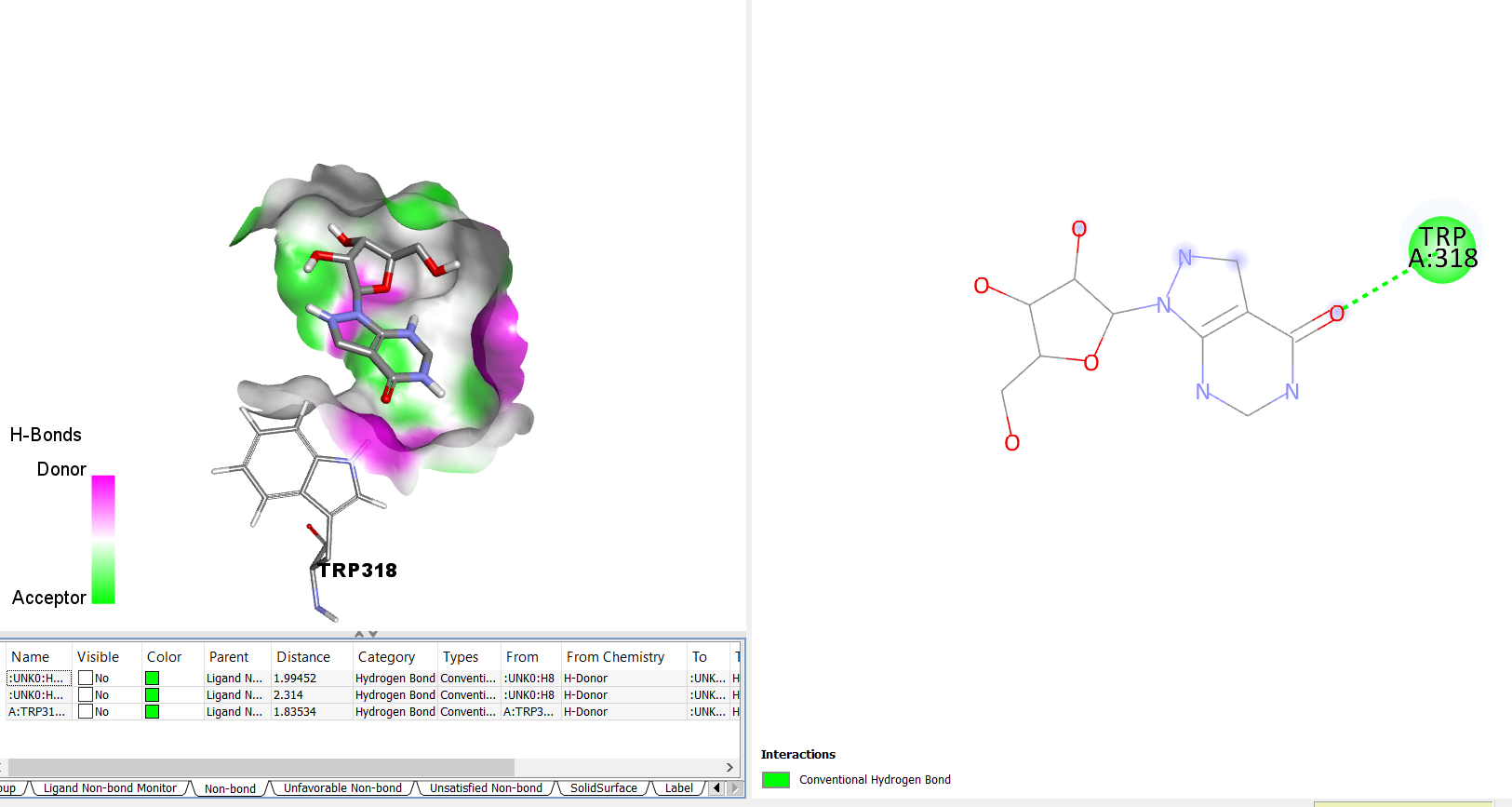 | 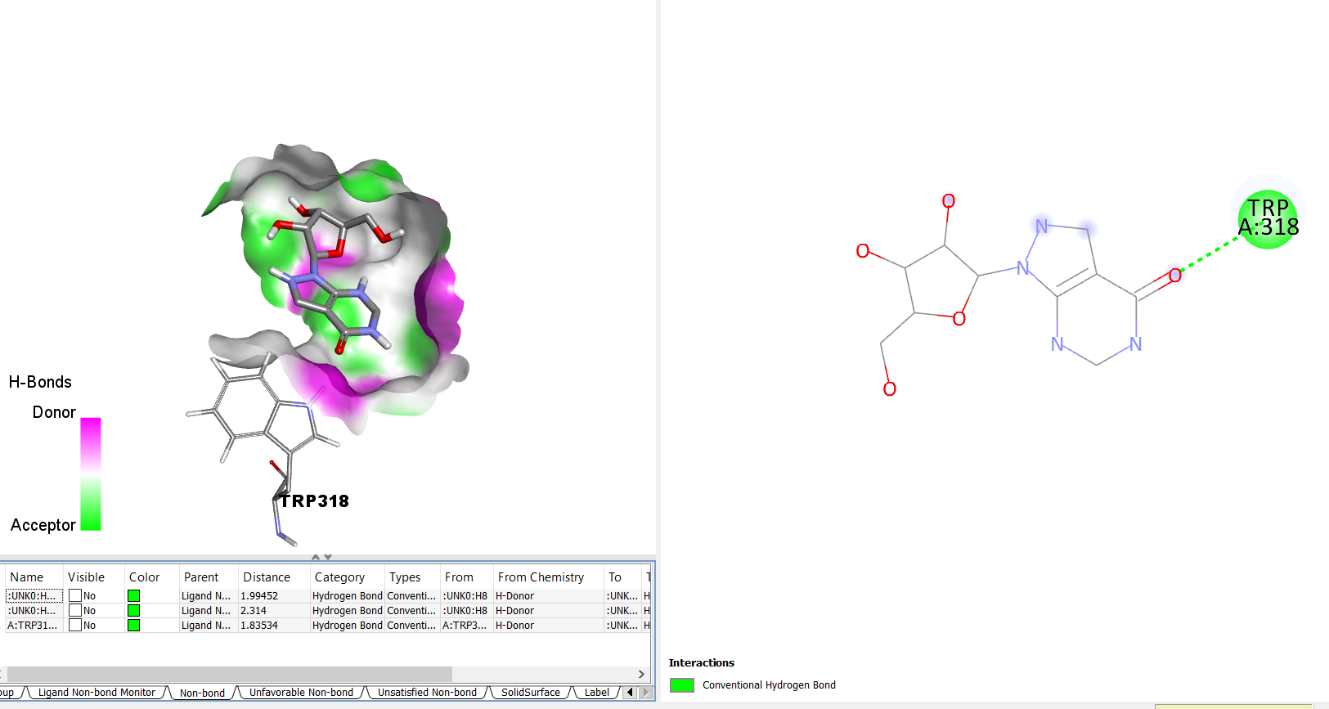 |
| Human MR1  [5UR1] | 1-(3,6,6-Trimethyl-1,6,7,7a-tetrahydrocyclopenta[c]pyran-1-yl)ethanone | 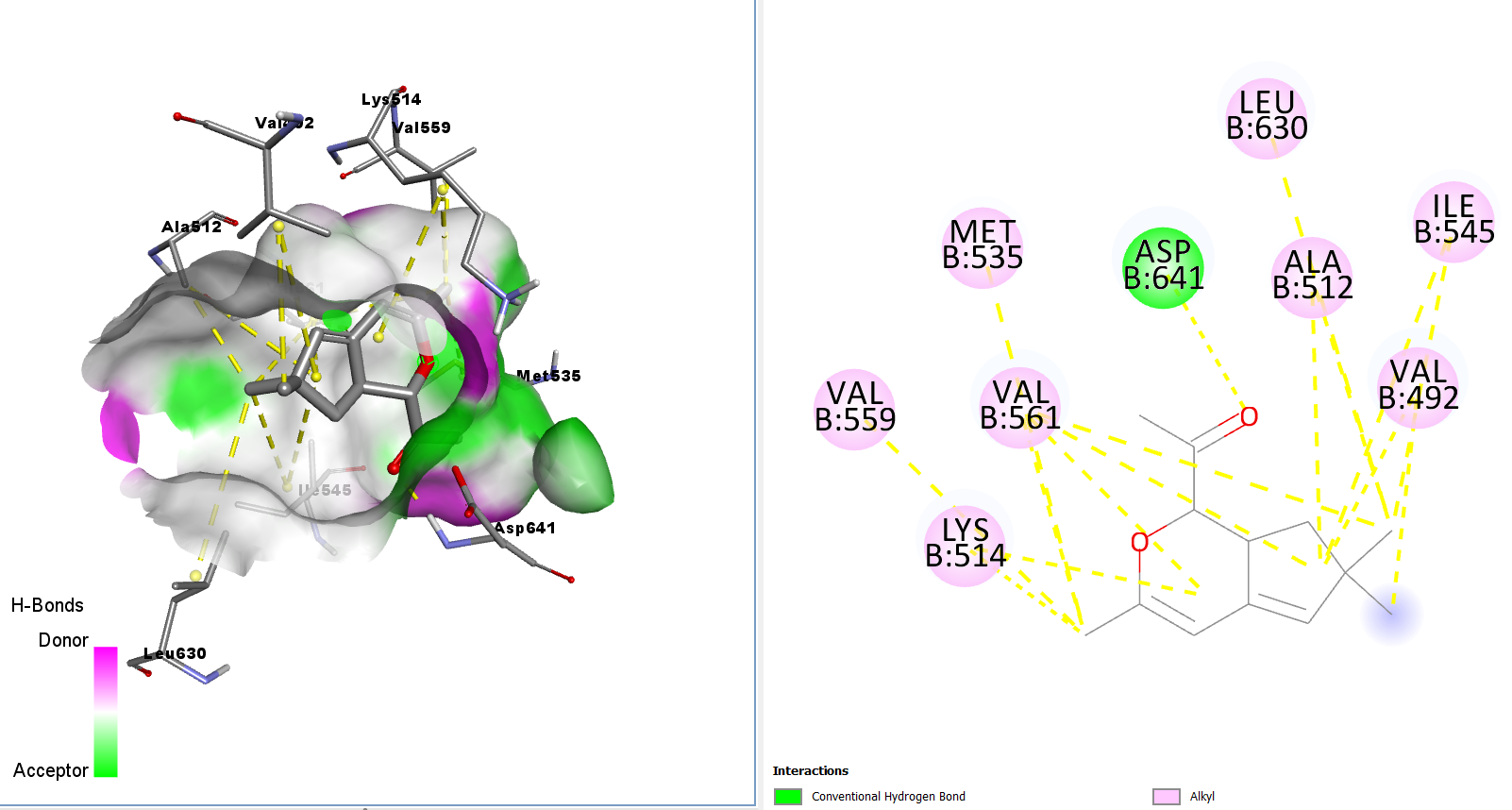 | 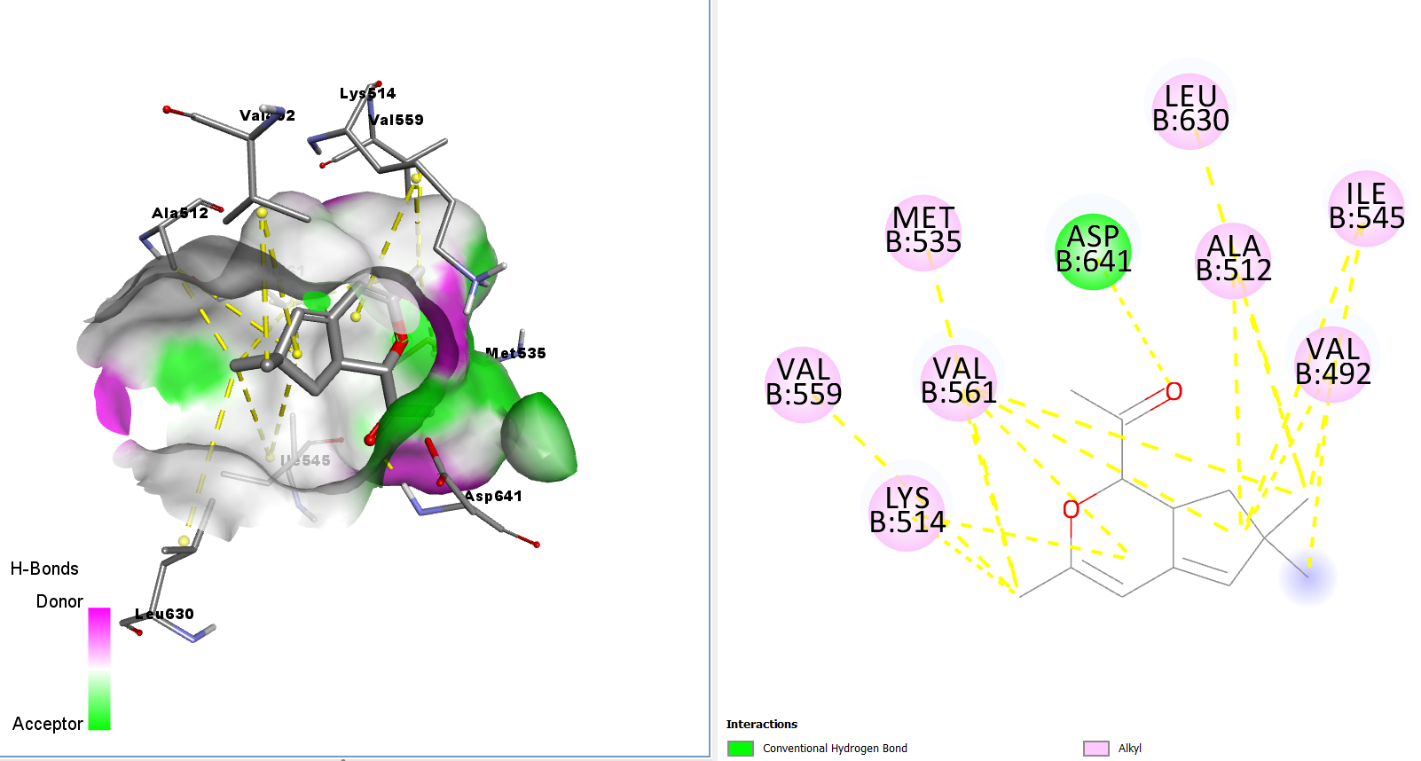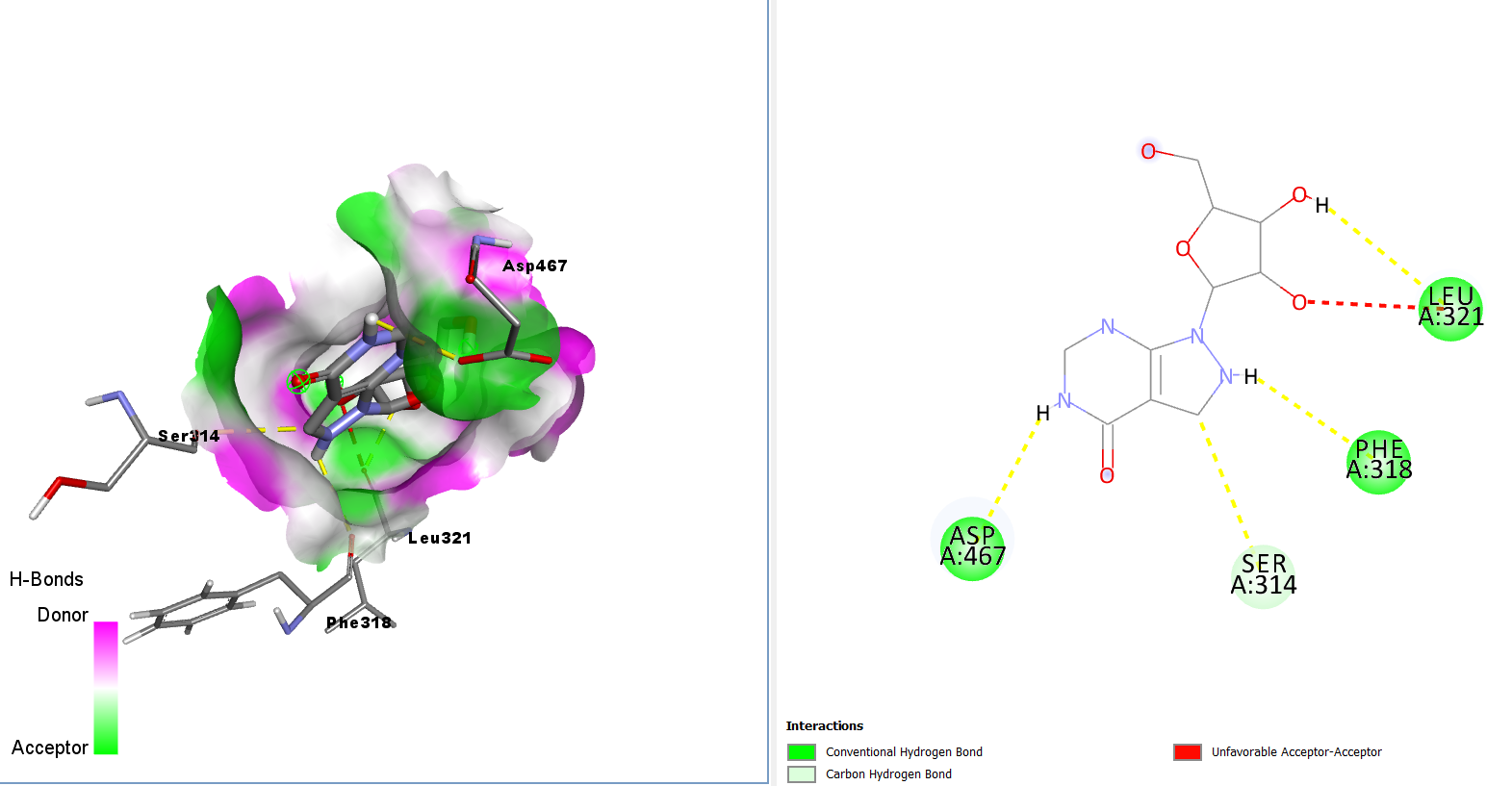 |
| Glutaminase 1 (GLS1)  [3VP1] | 4-Hydroxy-1H-pyrazolo[3,4-D]pyrimidine riboside | 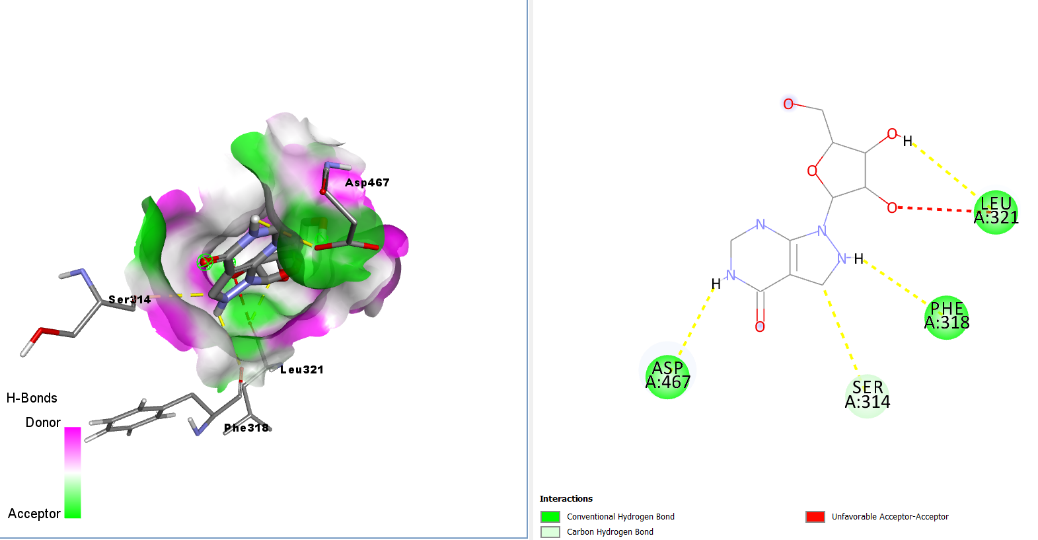 |  |
| Human GR  [1XAN] | 4-Hydroxy-1H-pyrazolo[3,4-D]pyrimidine riboside | 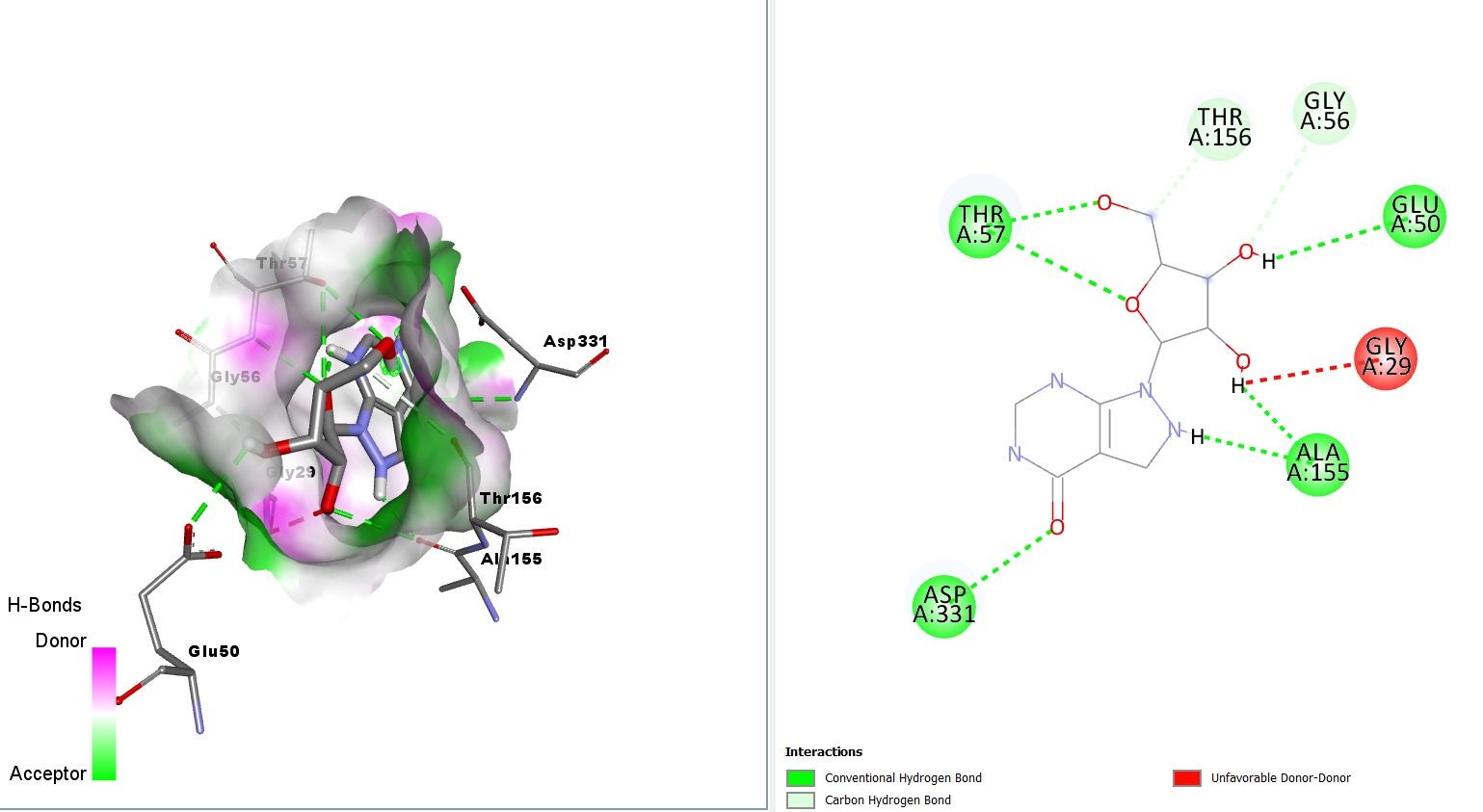 | 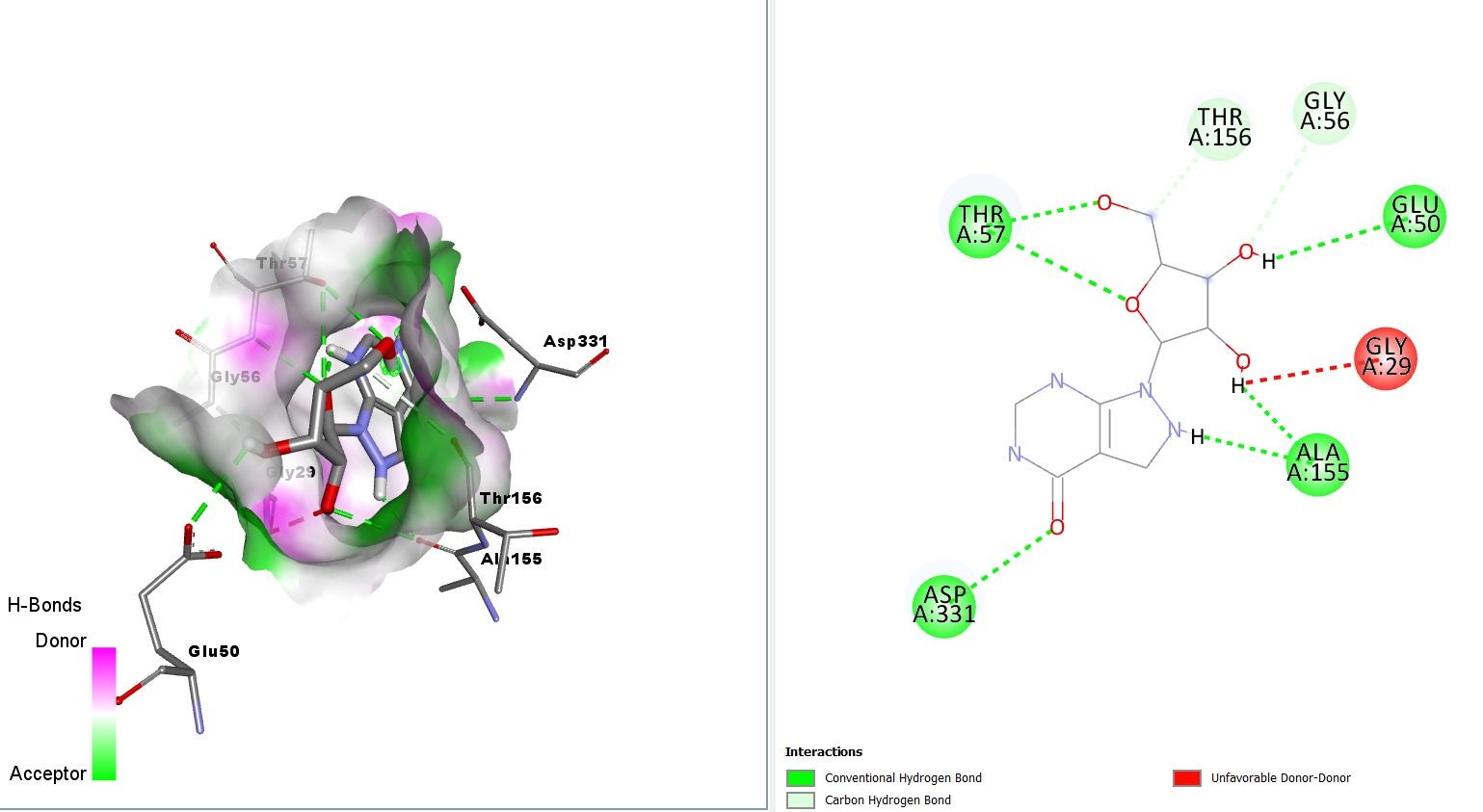 |
| Aldo-ketoreductase family 1 member C3 (AKR1C3)  [1S1P] | 1-(3,6,6-Trimethyl-1,6,7,7a-tetrahydrocyclopenta[c]pyran-1-yl)ethanone | 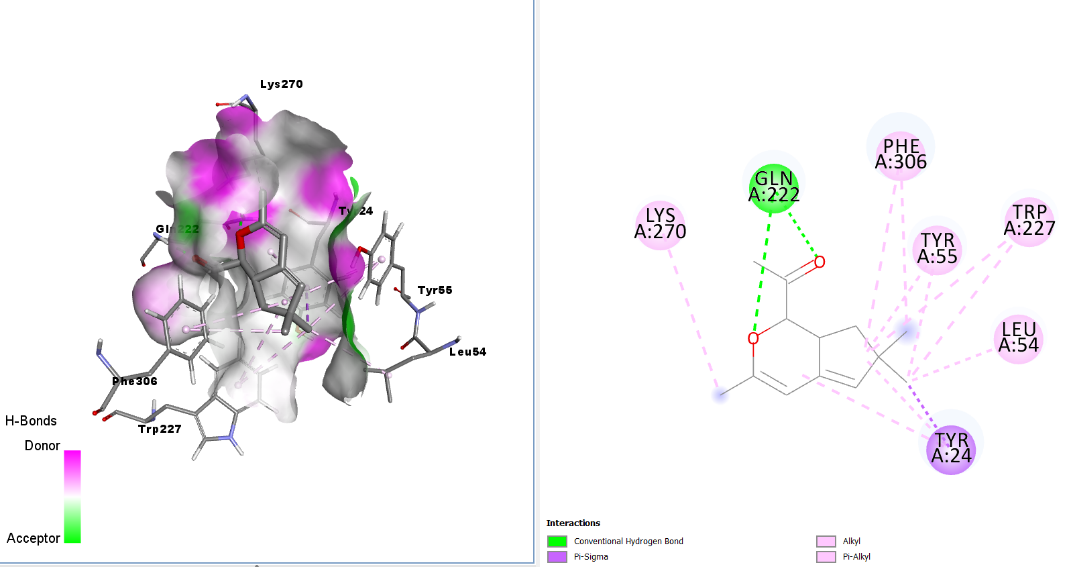 | 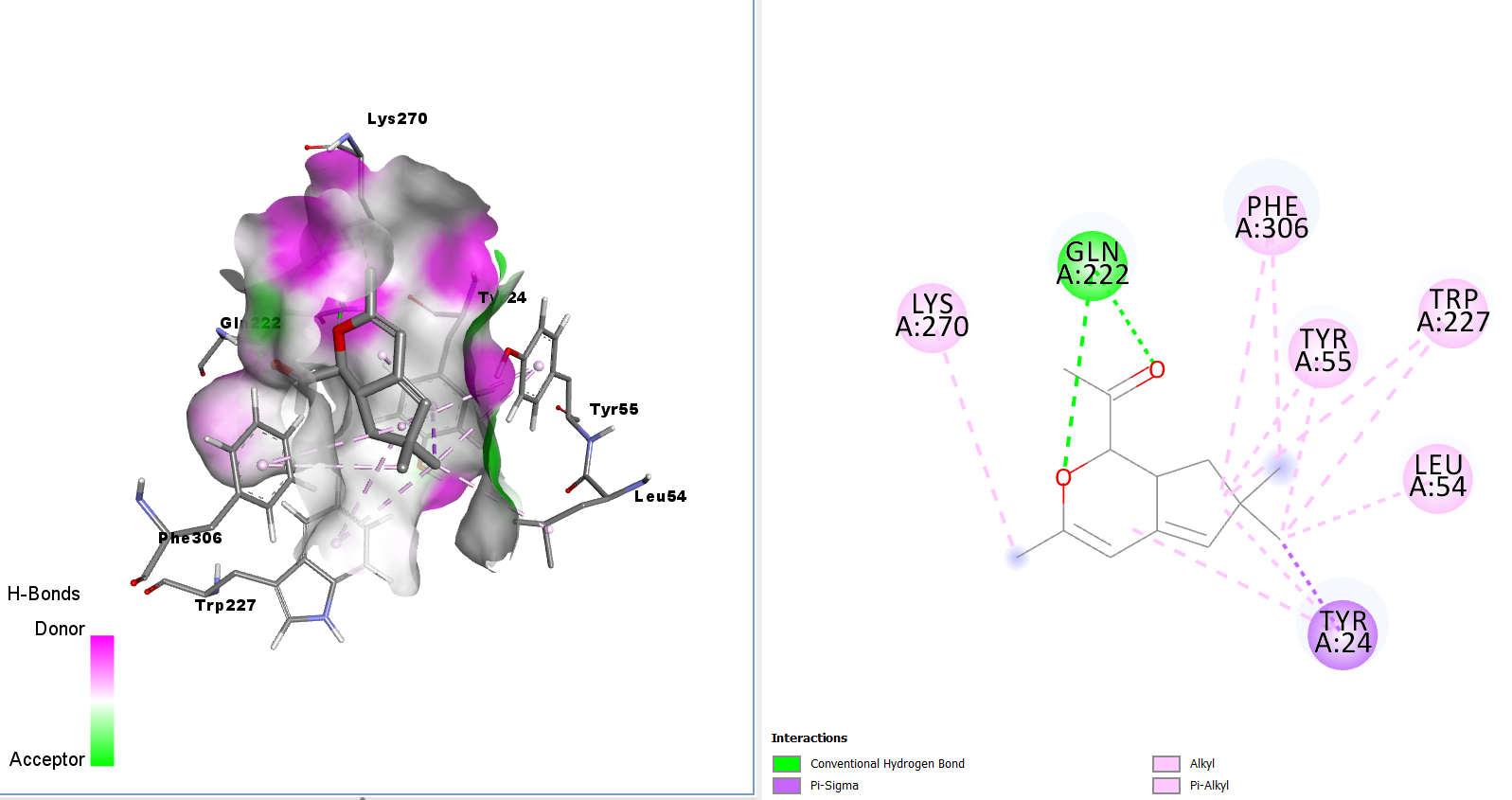 |
| Human erythrocyte catalase  [1DGH] | Maltose | 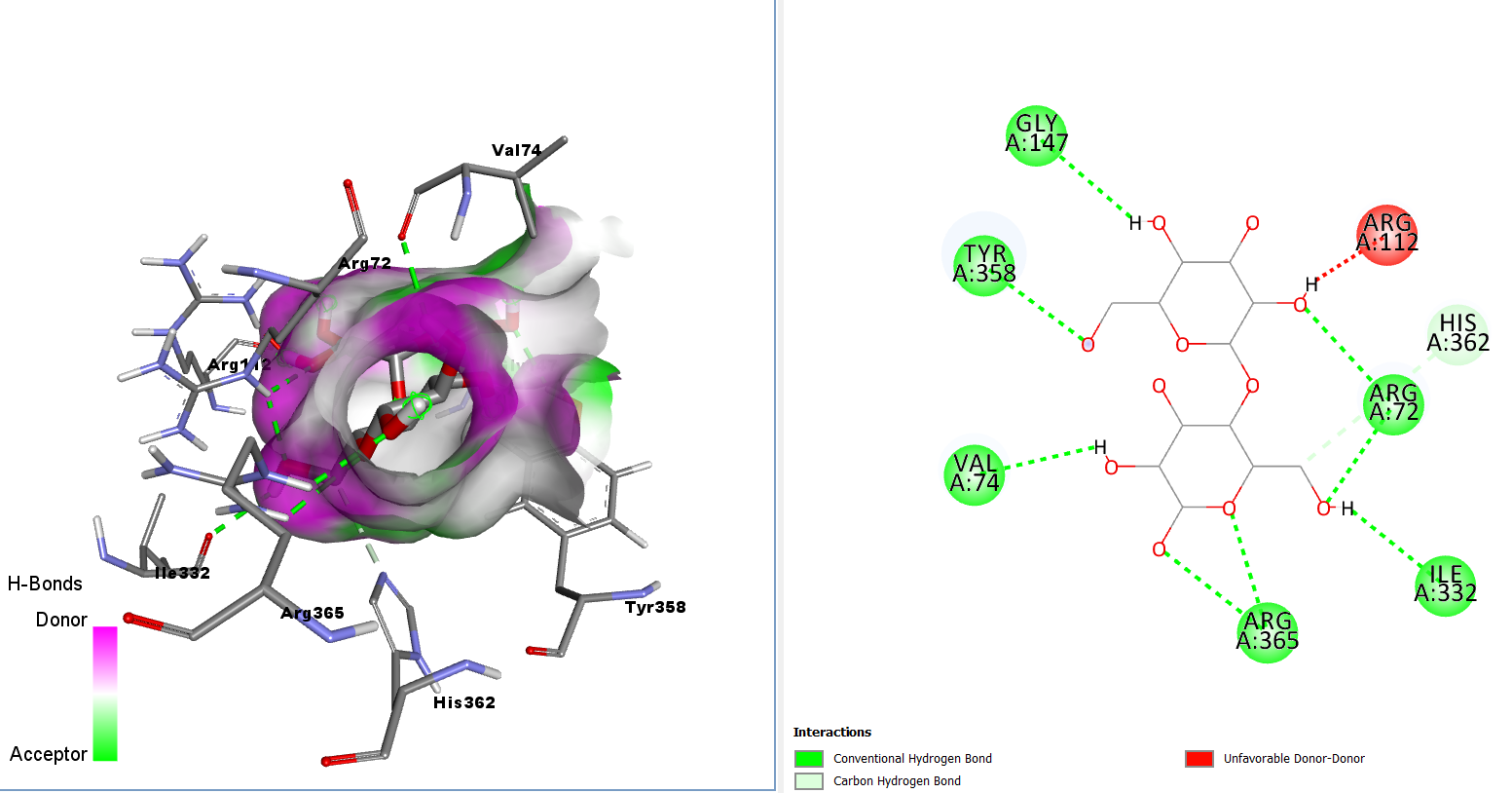 | 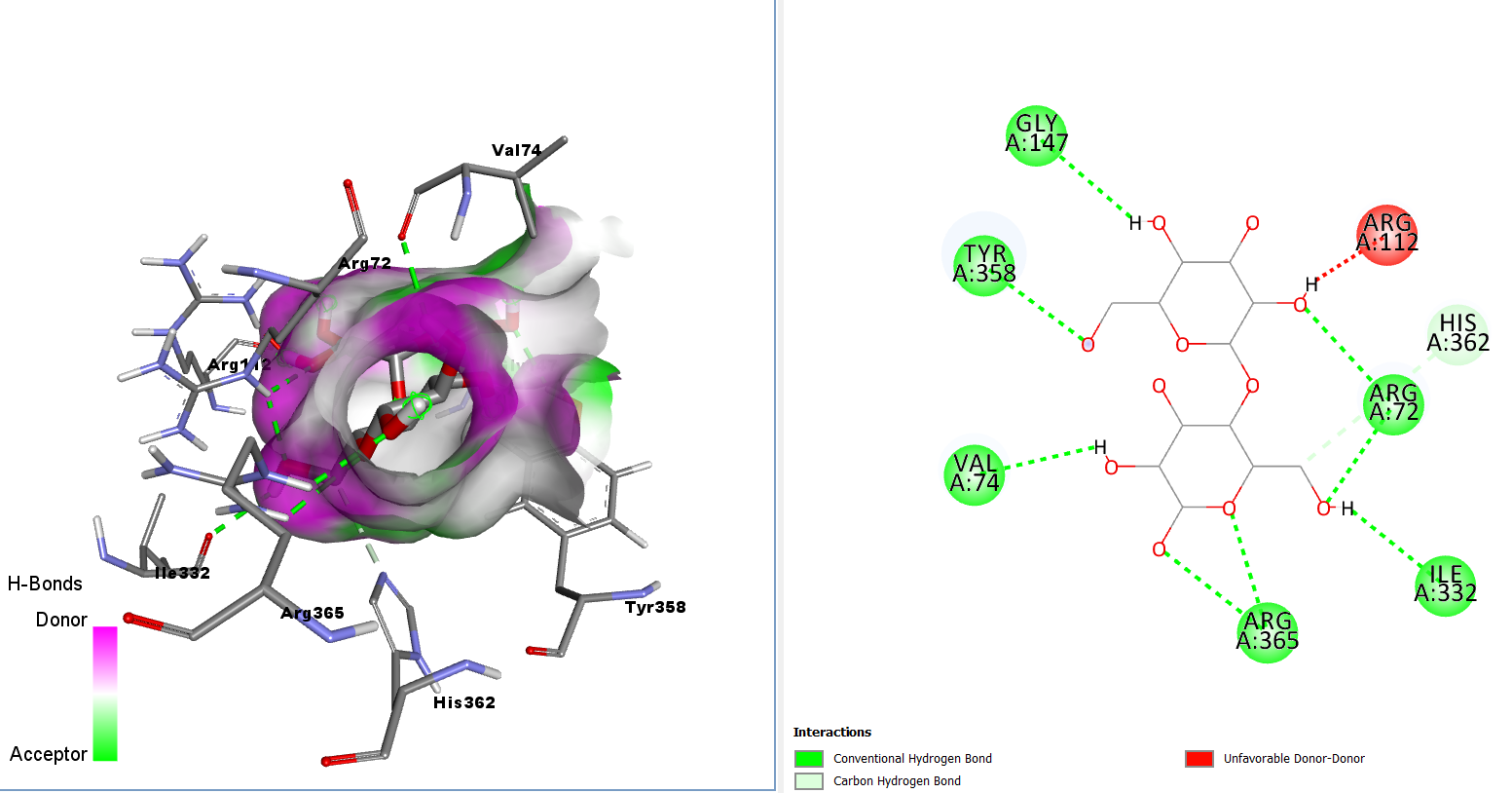 |

**Table 3** **Binding affinity of selected molecules with target proteins (PDB ID 2OYE,6COX, 5C1M)**

| **SL.No.** | **Compounds** | **Binding Affinity (Kcal/mol)** | | |
| --- | --- | --- | --- | --- |
|  |  | COX1  [2OYE] | COX2  [6COX] | Mu  [5C1M] |
| 1 | SL2_Hydroxyacetic acid, hydrazide_350536_pdb | -4.3 | -4.3 | -4 |
| 2 | SL3_Propanoic acid, 2-oxo-_1060_pdb | -4.1 | -3.8 | -3.9 |
| 3 | SL12_2-Methoxy-4-vinylphenol_332_pdb | -6.2 | -6 | -5.5 |
| 4 | SL18_1-(3,6,6-Trimethyl-1,6,7,7a-tetrahydrocyclopenta[c]pyran-1-yl)ethanone_605654_pdb | **-7.4** | **-8** | -7 |
| 5 | SL21_Benzeneacetic acid, 4-methoxy-, methyl ester_90266_pdb | -6.6 | -6.3 | -5.9 |
| 6 | SL25_Benzeneacetic acid, 3-methoxy-_15719_pdb | -6.4 | -6.4 | -5.9 |
| 7 | SL27_DL-Arabinitol_94154_pdb | -5.1 | -4.4 | -4 |
| 8 | SL28_2(4H)-Benzofuranone, 5,6,7,7a-tetrahydro-4,4,7a-trimethyl-, (R)-_6432173_pdb | -5.5 | -6.6 | -6.7 |
| 9 | SL29_Phenol, 4-ethenyl-2,6-dimethoxy-_35960_pdb | -6.2 | -5.8 | -5.8 |
| 10 | SL30_.alpha.-D-Glucopyranoside, methyl_64947_pdb | -5.4 | -5.6 | -4.9 |
| 11 | SL33_4-Hydroxy-1H-pyrazolo[3,4-D]pyrimidine riboside_135407110_pdb | **-7.4** | -7.5 | **-7.3** |
| 12 | SL36_Maltose_6255_pdb | -6.9 | -6.7 | -6.8 |
| 13 | SL42_(-)-Camphanic acid_719817_pdb | -5.3 | -6.7 | -6.3 |
| 14 | SL45_(E)-4-(3-Hydroxyprop-1-en-1-yl)-2-methoxyphenol_1549095_pdb | -6.4 | -6.6 | -5.9 |
| 15 | SL47_Loliolide_100332_pdb | -5.9 | -7.1 | -6.5 |
| 16 | SL55_Hexadecanoic acid, methyl ester_8181_pdb | -6.3 | -6.5 | -5.4 |
| 17 | SL56_l-(+)-Ascorbic acid 2,6-dihexadecanoate_54722209_pdb | -5.5 | -6.1 | -7.1 |
| 18 | SL59_trans-Sinapyl alcohol_5280507_pdb | -6.6 | -6.4 | -6 |
| 19 | SL62_8,11,14-Docosatrienoic acid, methyl ester_5364473_pdb | -6.8 | -6.8 | -6.3 |
| 20 | SL63_Phytol_5280435_pdb | -7 | -7.4 | -6.3 |
| 21 | SL64_Methyl stearate_8201_pdb | -6.1 | -6.7 | -5.6 |
| 22 | SL69_Hexadecanamide_69421_pdb | -6 | -6.8 | -5.5 |
| 23 | SL77_Palmitoleamide_56936054_pdb | -6.4 | -6.9 | -6.2 |

**Table 4 Binding affinity of selected molecules with target proteins (PDB ID 1DGH,1XAN)**

| **SL.No.** | **Compounds** | **Binding Affinity (Kcal/mol)** | |  |
| --- | --- | --- | --- | --- |
|  |  | Human erythrocyte catalase  [1DGH] | Human GR  [1XAN] | |
| 1 | SL2_Hydroxyacetic acid, hydrazide_350536_pdb | -4.8 | -4.3 | |
| 2 | SL3_Propanoic acid, 2-oxo-_1060_pdb | -4.5 | -4.3 | |
| 3 | SL12_2-Methoxy-4-vinylphenol_332_pdb | -6.5 | -5.7 | |
| 4 | SL18_1-(3,6,6-Trimethyl-1,6,7,7a-tetrahydrocyclopenta[c]pyran-1-yl)ethanone_605654_pdb | -8.2 | -7 | |
| 5 | SL21_Benzeneacetic acid, 4-methoxy-, methyl ester_90266_pdb | -6.8 | -6.2 | |
| 6 | SL25_Benzeneacetic acid, 3-methoxy-_15719_pdb | -6.9 | -6.3 | |
| 7 | SL27_DL-Arabinitol_94154_pdb | -5.3 | -5.3 | |
| 8 | SL28_2(4H)-Benzofuranone, 5,6,7,7a-tetrahydro-4,4,7a-trimethyl-, (R)-_6432173_pdb | -6.7 | -5.6 | |
| 9 | SL29_Phenol, 4-ethenyl-2,6-dimethoxy-_35960_pdb | -6.6 | -6.1 | |
| 10 | SL30_.alpha.-D-Glucopyranoside, methyl_64947_pdb | -6.3 | -6.3 | |
| 11 | SL33_4-Hydroxy-1H-pyrazolo[3,4-D]pyrimidine riboside_135407110_pdb | -8.5 | **-8.6** | |
| 12 | SL36_Maltose_6255_pdb | **-8.6** | -7.7 | |
| 13 | SL42_(-)-Camphanic acid_719817_pdb | -7.3 | -5.8 | |
| 14 | SL45_(E)-4-(3-Hydroxyprop-1-en-1-yl)-2-methoxyphenol_1549095_pdb | -6.9 | -6.5 | |
| 15 | SL47_Loliolide_100332_pdb | -6.8 | -6 | |
| 16 | SL55_Hexadecanoic acid, methyl ester_8181_pdb | -6.9 | -5.6 | |
| 17 | SL56_l-(+)-Ascorbic acid 2,6-dihexadecanoate_54722209_pdb | -8.2 | -7.2 | |
| 18 | SL59_trans-Sinapyl alcohol_5280507_pdb | -7 | -6.7 | |
| 19 | SL62_8,11,14-Docosatrienoic acid, methyl ester_5364473_pdb | -8 | -6.2 | |
| 20 | SL63_Phytol_5280435_pdb | -7.9 | -6.1 | |
| 21 | SL64_Methyl stearate_8201_pdb | -7.2 | -5.9 | |
| 22 | SL69_Hexadecanamide_69421_pdb | -7.1 | -5.4 | |
| 23 | SL77_Palmitoleamide_56936054_pdb | -7.2 | -5.4 | |

**Table 5 Binding affinity of selected molecules with target proteins (PDB ID 5UR1,1S1P)**

| **SL.No.** | **Compounds** | **Binding Affinity (Kcal/mol)** | |
| --- | --- | --- | --- |
|  |  | Human MR1  [5UR1] | Aldo-ketoreductase family 1 member C3 (AKR1C3)  [1S1P] |
| 1 | SL2_Hydroxyacetic acid, hydrazide_350536_pdb | -4 | -4.2 |
| 2 | SL3_Propanoic acid, 2-oxo-_1060_pdb | -3.7 | -4.2 |
| 3 | SL12_2-Methoxy-4-vinylphenol_332_pdb | -5.6 | -6.2 |
| 4 | SL18_1-(3,6,6-Trimethyl-1,6,7,7a-tetrahydrocyclopenta[c]pyran-1-yl)ethanone_605654_pdb | **-7.9** | **-8.8** |
| 5 | SL21_Benzeneacetic acid, 4-methoxy-, methyl ester_90266_pdb | -5.5 | -6.5 |
| 6 | SL25_Benzeneacetic acid, 3-methoxy-_15719_pdb | -5.7 | -6.6 |
| 7 | SL27_DL-Arabinitol_94154_pdb | -4.2 | -4.7 |
| 8 | SL28_2(4H)-Benzofuranone, 5,6,7,7a-tetrahydro-4,4,7a-trimethyl-, (R)-_6432173_pdb | -6 | -7.5 |
| 9 | SL29_Phenol, 4-ethenyl-2,6-dimethoxy-_35960_pdb | -5.8 | -6.5 |
| 10 | SL30_.alpha.-D-Glucopyranoside, methyl_64947_pdb | -4.7 | -5.9 |
| 11 | SL33_4-Hydroxy-1H-pyrazolo[3,4-D]pyrimidine riboside_135407110_pdb | -6.7 | -7.8 |
| 12 | SL36_Maltose_6255_pdb | -5.8 | -7.5 |
| 13 | SL42_(-)-Camphanic acid_719817_pdb | -5.9 | -7.3 |
| 14 | SL45_(E)-4-(3-Hydroxyprop-1-en-1-yl)-2-methoxyphenol_1549095_pdb | -5.8 | -6.6 |
| 15 | SL47_Loliolide_100332_pdb | -6.1 | -7.4 |
| 16 | SL55_Hexadecanoic acid, methyl ester_8181_pdb | -5.4 | -6.7 |
| 17 | SL56_l-(+)-Ascorbic acid 2,6-dihexadecanoate_54722209_pdb | -4.7 | -8.4 |
| 18 | SL59_trans-Sinapyl alcohol_5280507_pdb | -5.6 | -6.9 |
| 19 | SL62_8,11,14-Docosatrienoic acid, methyl ester_5364473_pdb | -6.2 | -8 |
| 20 | SL63_Phytol_5280435_pdb | -6.2 | -7.9 |
| 21 | SL64_Methyl stearate_8201_pdb | -5.8 | -6.9 |
| 22 | SL69_Hexadecanamide_69421_pdb | -5.8 | -7 |
| 23 | SL77_Palmitoleamide_56936054_pdb | -5.5 | -7 |

Table 6

| **SL.No.** | **Compounds** | **Binding Affinity (Kcal/mol)** |
| --- | --- | --- |
|  |  | Glutaminase 1 (GLS1)  [3VP1] |
| 1 | SL2_Hydroxyacetic acid, hydrazide_350536_pdb | -4.3 |
| 2 | SL3_Propanoic acid, 2-oxo-_1060_pdb | -4 |
| 3 | SL12_2-Methoxy-4-vinylphenol_332_pdb | -5.5 |
| 4 | SL18_1-(3,6,6-Trimethyl-1,6,7,7a-tetrahydrocyclopenta[c]pyran-1-yl)ethanone_605654_pdb | -6.9 |
| 5 | SL21_Benzeneacetic acid, 4-methoxy-, methyl ester_90266_pdb | -5.8 |
| 6 | SL25_Benzeneacetic acid, 3-methoxy-_15719_pdb | -6.1 |
| 7 | SL27_DL-Arabinitol_94154_pdb | -5.1 |
| 8 | SL28_2(4H)-Benzofuranone, 5,6,7,7a-tetrahydro-4,4,7a-trimethyl-, (R)-_6432173_pdb | -6.1 |
| 9 | SL29_Phenol, 4-ethenyl-2,6-dimethoxy-_35960_pdb | -5.6 |
| 10 | SL30_.alpha.-D-Glucopyranoside, methyl_64947_pdb | -5.4 |
| 11 | SL33_4-Hydroxy-1H-pyrazolo[3,4-D]pyrimidine riboside_135407110_pdb | **-7.3** |
| 12 | SL36_Maltose_6255_pdb | -6.5 |
| 13 | SL42_(-)-Camphanic acid_719817_pdb | -6.1 |
| 14 | SL45_(E)-4-(3-Hydroxyprop-1-en-1-yl)-2-methoxyphenol_1549095_pdb | -6 |
| 15 | SL47_Loliolide_100332_pdb | -6.5 |
| 16 | SL55_Hexadecanoic acid, methyl ester_8181_pdb | -5 |
| 17 | SL56_l-(+)-Ascorbic acid 2,6-dihexadecanoate_54722209_pdb | -6.3 |
| 18 | SL59_trans-Sinapyl alcohol_5280507_pdb | -6 |
| 19 | SL62_8,11,14-Docosatrienoic acid, methyl ester_5364473_pdb | -5.6 |
| 20 | SL63_Phytol_5280435_pdb | -5.9 |
| 21 | SL64_Methyl stearate_8201_pdb | -4.8 |
| 22 | SL69_Hexadecanamide_69421_pdb | -5.3 |
| 23 | SL77_Palmitoleamide_56936054_pdb | -5 |

**Table 7 Grid Box dimensions used for molecular docking**
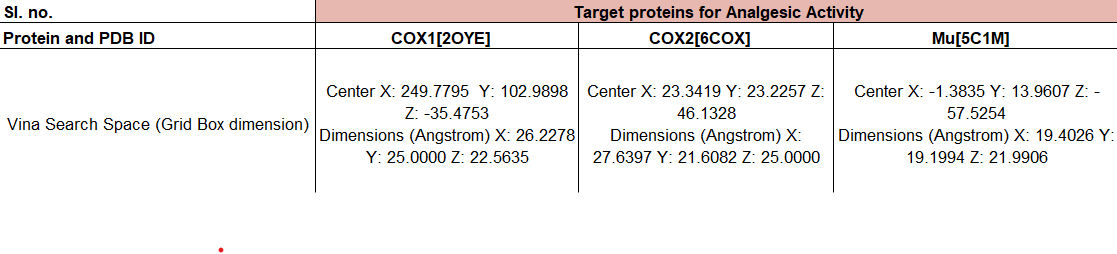


**Table 8 Grid Box dimensions used for molecular docking**


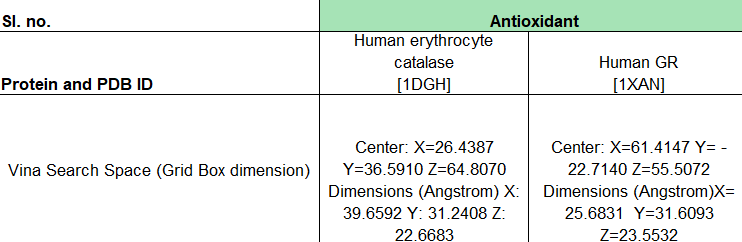


**Table 9 Grid Box dimensions used for molecular docking**


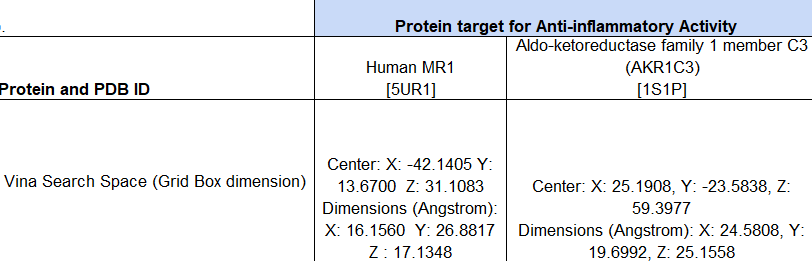


**Table 10 Grid Box dimensions used for molecular docking**


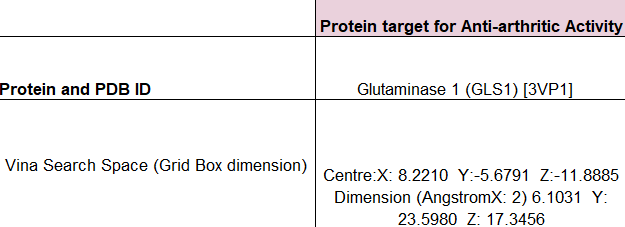

Supplement: Supplementary file 1 — Supporting Information 1 File S1: List of identified molecules by GC‐MS of the plant extract and molecular docking results of the selected molecules. [file BMRI-2026-8861594-s001.docx]
